# Integrated analysis of gene expression and copy number identified potential cancer driver genes with amplification-dependent overexpression in 1,454 solid tumors

**DOI:** 10.1038/s41598-017-00219-3

**Published:** 2017-04-04

**Authors:** Keiichi Ohshima, Keiichi Hatakeyama, Takeshi Nagashima, Yuko Watanabe, Kaori Kanto, Yuki Doi, Tomomi Ide, Yuji Shimoda, Tomoe Tanabe, Sumiko Ohnami, Shumpei Ohnami, Masakuni Serizawa, Koji Maruyama, Yasuto Akiyama, Kenichi Urakami, Masatoshi Kusuhara, Tohru Mochizuki, Ken Yamaguchi

**Affiliations:** 1 Medical Genetics Division, Shizuoka Cancer Center Research Institute, Shizuoka, 411-8777 Japan; 2 Cancer Diagnostics Research Division, Shizuoka Cancer Center Research Institute, Shizuoka, 411-8777 Japan; 3 SRL, Inc., Tokyo, 163-0409 Japan; 4 Drug Discovery and Development Division, Shizuoka Cancer Center Research Institute, Shizuoka, 411-8777 Japan; 5 Experimental Animal Facility, Shizuoka Cancer Center Research Institute, Shizuoka, 411-8777 Japan; 6 Immunotherapy Division, Shizuoka Cancer Center Research Institute, Shizuoka, 411-8777 Japan; 7 Region Resources Division, Shizuoka Cancer Center Research Institute, Shizuoka, 411-8777 Japan; 8 Shizuoka Cancer Center Hospital and Research Institute, Shizuoka, 411-8777 Japan

## Abstract

Identification of driver genes contributes to the understanding of cancer etiology and is imperative for the development of individualized therapies. Gene amplification is a major event in oncogenesis. Driver genes with tumor-specific amplification-dependent overexpression can be therapeutic targets. In this study, we aimed to identify amplification-dependent driver genes in 1,454 solid tumors, across more than 15 cancer types, by integrative analysis of gene expression and copy number. Amplification-dependent overexpression of 64 known driver oncogenes were found in 587 tumors (40%); genes frequently observed were *MYC* (25%) and *MET* (18%) in colorectal cancer; *SKP2* (21%) in lung squamous cell carcinoma; *HIST1H3B* (19%) and *MYCN* (13%) in liver cancer; *KIT* (57%) in gastrointestinal stromal tumors; and *FOXL2* (12%) in squamous cell carcinoma across tissues. Genomic aberrations in 138 known cancer driver genes and 491 established fusion genes were found in 1,127 tumors (78%). Further analyses of 820 cancer-related genes revealed 16 as potential driver genes, with amplification-dependent overexpression restricted to the remaining 22% of samples (327 tumors) initially undetermined genetic drivers. Among them, *AXL*, which encodes a receptor tyrosine kinase, was recurrently overexpressed and amplified in sarcomas. Our studies of amplification-dependent overexpression identified potential drug targets in individual tumors.

## Introduction

Driver genes involved in oncogenesis are generated by genomic alterations, including point mutations, insertions, deletions, translocations, and gene amplifications^1^. Driver genes are considered “druggable” targets, using molecular targeted therapies, in which molecules selectively bind protein products translated from genes with mutations, or expressed from amplified genes or fusion genes, to inhibit their oncogenic activities. Examples of such therapies include EGFR inhibitors, which target point mutations in *EGFR* ^2^, anti-HER2 antibodies recognizing the product of the *ERBB2* gene amplification^3^, and ALK inhibitors, which target the *EML4-ALK* fusion gene^4^. Identification of novel driver genes has been accelerated by recent developments in DNA sequencing technologies, including next-generation sequencing (NGS), particularly in the context of large-scale cancer genomic studies, such as The Cancer Genome Atlas (TCGA, http://cancergenome.nih.gov/). To date, the number of known driver genes varies from 138^1^ to 602 genes, which are listed in the database of the Cancer Gene Census and has been updated from the initial 291 genes^5^. The majority of known driver genes are based on mutations.

Gene amplification is a relatively frequent event in cancer genomes; however, genomic amplification is not always accompanied by elevated gene expression^6^. Conversely, overexpression is requisite for amplified genes to function as driver alterations. Since they are overexpressed, genes with amplification-dependent overexpression are ideal targets for molecular targeting therapies using antibodies. Thus, integration of copy number variations (CNVs) and mRNA expression levels to identify candidate driver genes have been reported^7, 8^, and the development of TCGA datasets has accelerated the search for driver genes using *in silico* methods^7^.

With the aim of introducing individualized medicine for cancer patients in the future, Shizuoka Cancer Center launched Project HOPE in 2014, which is based on the multi-omics analyses including whole exome sequencing (WES) and gene expression profiling (GEP)^9^. In this study, we describe gene expression and copy number analysis to determine the involvement of amplification-based driver genes in 1,454 tumors. Coupled with the analysis of 138 mutation-based and 491 fusion-based driver genes, we further explored candidate amplification-based driver genes by examination of a wider candidate cancer-related gene dataset in those tumors with driver origins undetermined by our initial analyses, resulting in the identification of 16 additional potential amplification-based driver genes.

## Results

### GEP of 64 driver oncogenes in 1,454 solid tumors

**Figure 1:**
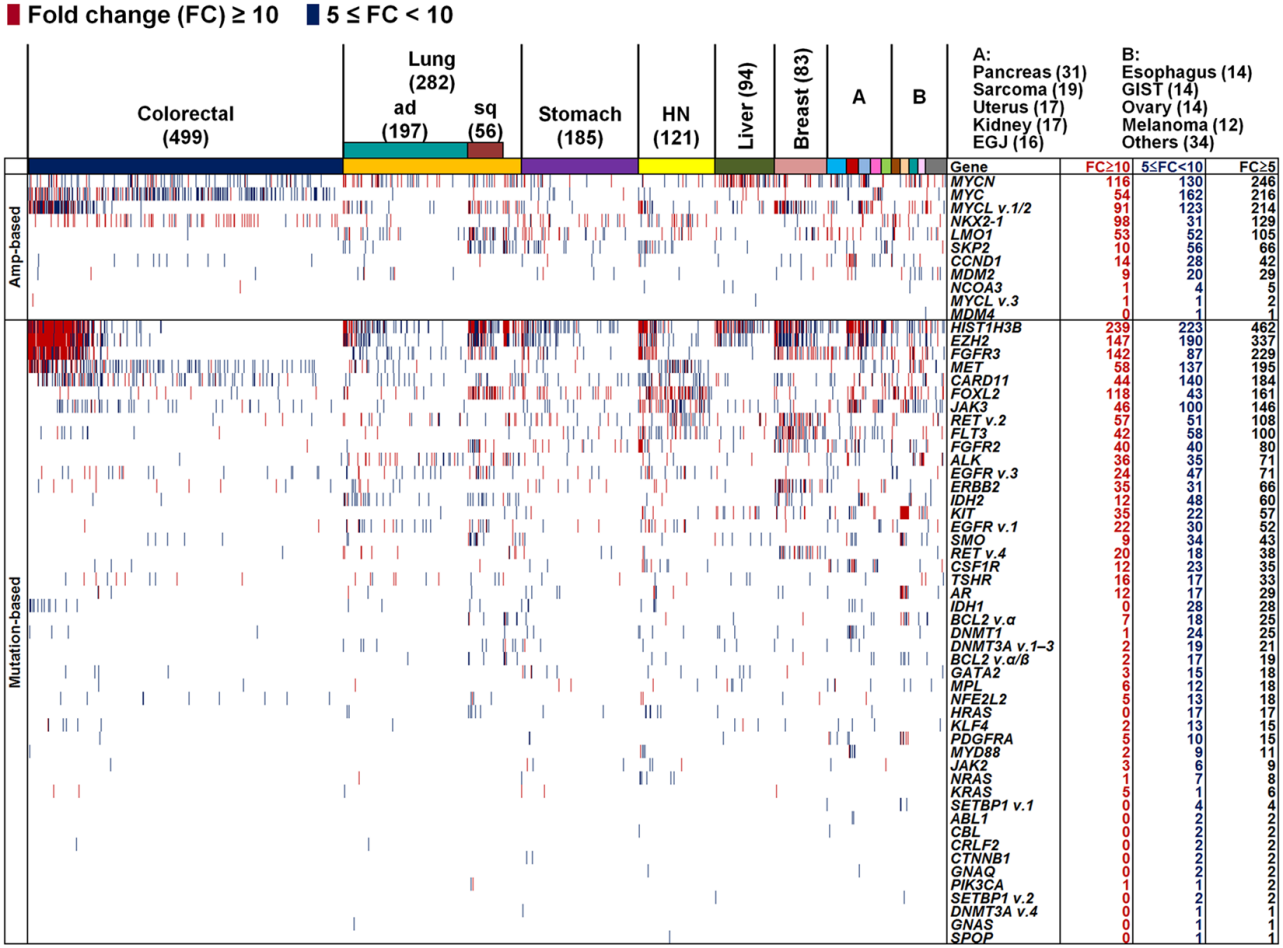
Elevated expression levels of 64 oncogenes in 1,454 solid tumors across 15 cancer types. Cased with strong (fold change ≥10) and moderate (5≤ fold change <10) overexpression are indicated by dark red and dark blue bars, respectively. The two groups of oncogenes, 10 amplification-based (Amp-based), and 54 mutation-based, oncogenes, are indicated on the left. Since some probes recognize multiple splice variants, the number of microarray probes to identify the 64 oncogenes was 71, and there were 13 probes among these corresponding to 12 genes which showed no elevated expression in any tumors, which are not shown. The numbers on the right indicate the number of samples with strong (fold change ≥10) and moderate (5≤ fold change <10) overexpression, as well as the sum of these samples (fold change ≥5). As indicated at the top, samples are arranged by tumor type, including colorectal, lung, stomach, head and neck (HN), liver, breast, pancreas, sarcoma, uterus, kidney, esophagogastric junction (EGJ), esophagus, gastrointestinal stromal tumor (GIST), ovary, melanoma, and other types of tumors. The number of samples of each type of tumor is indicated in parentheses.

Gene expression analysis is requisite to confirm the status of tumor-specific genomic alterations, including mutations and amplifications, as actionable cancer driver genes. Thus, to determine potential driver carcinogenic genetic changes in 1,454 solid tumors (Supplementary Fig. 1), we first investigated the mRNA expression levels of 64 known driver oncogenes (Supplementary Table 1)^1^. Among the 64 oncogenes, 10 genes (*CCND1*, *LMO1*, *MDM2*, *MDM4*, *MYC*, *MYCL*, *MYCN*, *NCOA3*, *NKX2-1*, and *SKP2*) were categorized as amplification-based oncogenes^1^. The remaining 54 genes were mutation-based oncogenes, primary affected by base substitutions, intragenic insertions, and deletions. We assessed the fold change (FC) in expression levels between tumors and corresponding matched normal tissues by microarray analysis; genes with expression levels increased ≥5-fold in tumor tissues were defined as overexpressed. Among the 10 amplification-based oncogenes, expression levels of *MYC* family genes, including *MYC*, *MYCL*, and *MYCN*, varied among tumor tissue samples (Fig. 1 and Supplementary Fig. 2). A relatively high frequency of overexpression of *MYC* was observed in kidney (48%) and colorectal (37%) cancers; of *MYCL* (variants 1 and 2) in uterine (58%) and breast (40%) cancers; and of *MYCN* in uterine (58%), liver (44%), and ovarian (43%) cancers. In addition, *CCND1* was frequently overexpressed in sarcoma (53%), 79% of which (42% of total sarcoma samples) showed a ≥10-fold increase in expression.

Among the 54 mutation-based oncogenes, *HIST1H3B* and *EZH2*, which are involved in chromatin structure and remodeling, were overexpressed in 32% and 23%, respectively, of various types of tumors (Fig. 1 and Supplementary Fig. 2). These genes, along with *FGFR3*, appear to be co-overexpressed in colorectal cancer. Genes frequently overexpressed in specific tumors were as follows: *ALK* in melanoma (50%) as previously reported^10^; *RET* [reviewed in ref. 11] (37% for variant 2 and 35% for variant 4), *FLT3* (44%) and *ERBB2* ^12^ (34%) in breast cancer; *CSF1R* (47%), *DNMT1* (42%), and *MYD88* (31%) in sarcoma; *KIT* ^13, 14^ (100%), *AR* (71%), *SMO* (50%), *PDGFRA* ^14^ (43%), and *BCL2* ^15^ (variant alpha) (42%) in gastrointestinal stromal tumors (GISTs). Overall, these data revealed different levels of expression of driver oncogenes across various cancer types.

### Oncogenic driver gene amplification of 64 known oncogenes assessed by integrative analysis of GEP and CNVs

**Figure 2:**
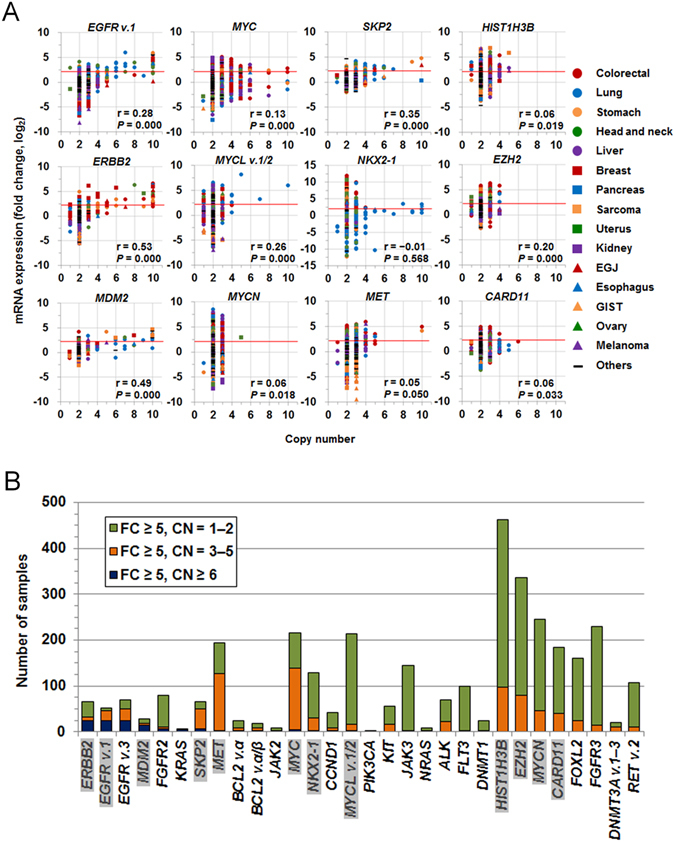
Gene expression and copy number of 64 oncogenes. (**A**) Gene expression levels of 12 oncogenes, including *EGFR* variant 1, *ERBB2*, *MDM2*, *MYC*, *MYCL* variants 1 and 2, *MYCN*, *SKP2*, *NKX2-1*, *MET*, *HIST1H3B*, *EZH2*, and *CARD11*, were linked to the genomic copy number. Pearson’s correlation coefficient (r with *P*-value) between copy number and mRNA expression is indicated at the bottom right. Red horizontal bars indicate 5-fold on the log_2_ scale. (**B**) The relationship between gene overexpression and degree of genomic copy number gain. Tumors in which oncogenes were overexpressed by ≥5-fold were divided into three groups: those with high-level genomic amplification (copy number ≥6; dark blue bars), those with moderate genomic amplification (copy number 3–5; orange bars), and those without genomic amplification (copy number 1–2; light green bars). There were no cases with a copy number of zero among the indicated genes. Genes highlighted on the bottom correspond to the 12 oncogenes exhibited in panel A.

Overexpressed oncogenes with amplified respective chromosomal loci are candidate driver genes. In contrast, genomically amplified oncogenes without corresponding overexpression appear to have no, or less, involvement in oncogenesis. Thus, we next integrated the gene expression data for the 64 known oncogenes with the corresponding genomic copy number results, to predict oncogenic driver gene amplifications. As a primary analysis, we selected 12 genes from the 64 oncogenes. These include nine genes frequently overexpressed with copy number gain, including six amplification-based oncogenes (*MYC*, *MYCL*, *MYCN*, *MDM2*, *NKX2-1*, and *SKP2*) and three mutation-based oncogenes (*HIST1H3B*, *EZH2*, and *CARD11*) (Supplementary Fig. 3), and three mutation-based oncogenes, including *ERBB2*, *EGFR*, and *MET*, exhibiting amplifications associated with cancer^6^. As shown in Fig. 2A, the numbers of copies of these 12 oncogenes were diverse. Thus, we defined the degree of amplification by copy number as follows: genes with copy number ≥6 were defined as being highly amplified, while those with copy numbers of 3, 4, and 5 were defined as being moderately amplified. Next, the 12 oncogenes were divided into two groups based on their copy numbers in samples with ≥5-fold overexpression (Fig. 2A,B). One group contained genes where overexpression was frequent among those with high genomic amplification (copy number ≥6), and included *EGFR* (variant 1), *ERBB2*, and *MDM2*. The other group, contained the remaining nine genes, whose overexpression was frequent among samples with moderate genomic amplification (copy number 3–5), including *MYC*, *MYCL* (variants 1 and 2), *MYCN*, *SKP2*, *NKX2-1*, *MET*, *HIST1H3B*, *EZH2*, and *CARD11*. In addition to *EGFR* variant 1, *ERBB2*, and *MDM2*, samples overexpressing *FGFR2*, *KRAS*, and *EGFR* variant 3 were abundant among those high-level genomic amplification of these genes, specifically in stomach cancer (*FGFR2*), colorectal and stomach cancer (*KRAS*), and lung, and head and neck cancer (*EGFR* variant 3) samples (Fig. 2B and Supplementary Fig. 3). When samples overexpressing *EGFR* variants 1 and 3 included samples with both high and moderate-levels of genomic amplification, samples in which both *EGFR* variants were overexpressed with genomic amplification were abundant in lung, and head and neck cancer samples (Fig. 2B and Supplementary Fig. 4). Interestingly, samples in which only *EGFR* variant 3 was overexpressed with genomic amplification were abundant in colorectal cancer samples. *EGFR* variant 3 mRNA translates a soluble EGFR protein, p60 (isoform C), lacking transmembrane and tyrosine kinase domains, whereas *EGFR* variant 1 mRNA translates the full-length p170 EGFR (isoform A)^16, 17^. Because the formation of inactive heterodimers between different isoforms competitively prevents the formation of functional holoreceptors^18^, this result suggested that oncogenesis involving the EGFR pathway differs in samples with or without overexpressing *EGFR* variant 3 exhibiting genomic amplification.

Using Pearson correlation coefficient, *ERBB2* and *MDM2* showed moderate relationships between copy number and FC (r = 0.53 and 0.49, respectively), whereas *SKP2* (r = 0.35), *EGFR* variant 1 (r = 0.28), *MYCL* variants 1 and 2 (r = 0.26), and *EZH2* (r = 0.20) were in the range between 0.2 and 0.4, indicating weak relationships. In particular, the majority of samples with high-level amplification of *MYC* were not overexpressed (r = 0.13), suggesting that these amplifications were passenger-like. Thus, we investigated the frequency of amplification in samples demonstrating overexpression (Table 1). Strong to moderate correlations between overexpression and amplification (defined as frequencies of ≥50%) were observed for *EGFR* (88% and 70% for variants 1 and 3, respectively), *SKP2* (76%), *MET* (65%), *MYC* (64%), *MDM2* (62%), *DNMT3A* (52%), and *ERBB2* (50%). For these eight genes, Pearson correlation coefficient between copy number and FC among the samples overexpressed with FC ≥5 demonstrated moderate and weak relationships in *EGFR* variant 1 (r = 0.52) and *MYC* (r = 0.33), respectively (Supplementary Fig. 5), which increased from those obtained from all 1,454 samples (r = 0.28 and 0.13, respectively). This result indicates that overexpression with FC ≥5 of *EGFR* variant 1 and *MYC* is frequently involved in copy number gain. In contrast, weak correlations (≤10% frequency) were observed for *IDH2* (10%), *RET* (10% and 8% for variants 2 and 4, respectively), *DNMT1* (8%), *MYCL* variants 1 and 2 (8%), *FGFR3* (6%), *LMO1* (4%), *FLT3* (3%), *JAK3* (1%), and *AR* (0%).

**Table 1:** Comparison of the number of samples in which oncogenes were overexpressed and amplified with samples in which oncogenes were only overexpressed.

Gene	Oncogene type	Pearson’s correlation coefficient between FC and CN among in 1,454 samples		Number of samples				Ratio (%)		
		r	*P*-value	Overexpressed (FC ≥5, A)	Overexpressed and amplified (FC ≥5, CN ≥3, B)	Overexpressed and highly amplified (FC ≥5, CN ≥6, C)	Overexpressed and moderately amplified (FC ≥5, CN = 3–5, D)	(B) to (A)	(C) to (A)	(D) to (A)
*MYCN*	Amplification-based	0.06	0.018	246	47	0	47	19	0	19
*MYC*	Amplification-based	0.13	0.000	216	139	4	135	64	2	63
*MYCL v.1/2*	Amplification-based	0.26	0.000	214	17	2	15	8	1	7
*NKX2-1*	Amplification-based	−0.01	0.568	129	31	3	28	24	2	22
*LMO1*	Amplification-based	0.01	0.798	105	4	0	4	4	0	4
*SKP2*	Amplification-based	0.35	0.000	66	50	7	43	76	11	65
*CCND1*	Amplification-based	0.12	0.000	42	8	3	5	19	7	12
*MDM2*	Amplification-based	0.49	0.000	29	18	14	4	62	48	14
*HIST1H3B*	Mutation-based	0.06	0.019	462	97	0	97	21	0	21
*EZH2*	Mutation-based	0.20	0.000	337	80	0	80	24	0	24
*FGFR3*	Mutation-based	−0.03	0.212	229	14	0	14	6	0	6
*MET*	Mutation-based	0.05	0.050	195	127	2	125	65	1	64
*CARD11*	Mutation-based	0.06	0.033	184	41	0	41	22	0	22
*FOXL2*	Mutation-based	0.08	0.001	161	25	0	25	16	0	16
*JAK3*	Mutation-based	0.12	0.000	146	2	1	1	1	1	1
*RET v.2*	Mutation-based	0.07	0.012	108	11	0	11	10	0	10
*FLT3*	Mutation-based	−0.03	0.325	100	3	1	2	3	1	2
*FGFR2*	Mutation-based	0.37	0.000	80	10	7	3	13	9	4
*ALK*	Mutation-based	0.01	0.813	71	23	1	22	32	1	31
*EGFR v.3*	Mutation-based	0.12	0.000	71	50	24	26	70	34	37
*ERBB2*	Mutation-based	0.53	0.000	66	33	24	9	50	36	14
*IDH2*	Mutation-based	0.07	0.006	60	6	0	6	10	0	10
*KIT*	Mutation-based	0.14	0.000	57	16	1	15	28	2	26
*EGFR v.1*	Mutation-based	0.28	0.000	52	46	24	22	88	46	42
*SMO*	Mutation-based	0.04	0.110	43	5	0	5	12	0	12
*RET v.4*	Mutation-based	0.01	0.674	38	3	0	3	8	0	8
*CSF1R*	Mutation-based	−0.03	0.210	35	4	0	4	11	0	11
*TSHR*	Mutation-based	0.05	0.050	33	5	0	5	15	0	15
*AR*	Mutation-based	N/A*	N/A	29	0	0	0	0	0	0
*IDH1*	Mutation-based	0.12	0.000	28	4	0	4	14	0	14
*BCL2 v.α*	Mutation-based	0.32	0.000	25	9	2	7	36	8	28
*DNMT1*	Mutation-based	0.15	0.000	25	2	1	1	8	4	4
*DNMT3A v.1–3*	Mutation-based	0.30	0.000	21	11	0	11	52	0	52
*BCL2 v.α/β*	Mutation-based	0.39	0.000	19	9	2	7	47	11	37
*GATA2*	Mutation-based	−0.06	0.030	18	3	0	3	17	0	17
*MPL*	Mutation-based	0.10	0.000	18	3	0	3	17	0	17
*NFE2L2*	Mutation-based	0.05	0.053	18	4	0	4	22	0	22
*HRAS*	Mutation-based	0.09	0.001	17	3	0	3	18	0	18
*KLF4*	Mutation-based	0.11	0.000	15	2	0	2	13	0	13
*PDGFRA*	Mutation-based	0.01	0.739	15	2	0	2	13	0	13
*MYD88*	Mutation-based	0.08	0.002	11	2	0	2	18	0	18

^*^N/A: data was not available due to the lack of copy number alteration among samples.

The frequency of amplification-dependent overexpression, in which overexpression was accompanied by either high or moderate levels of genomic amplification, was calculated for individual tumor types (Table 2). For seven types of tumors, where ≥50 samples were available, genes identified as amplified and overexpressed with frequency of ≥10% were *MYC* (25%) and *MET* (18%) in colorectal cancer; *SKP2* (21%), *FOXL2* (18%), *EGFR* (16% and 14% for variants 3 and 1, respectively) in lung squamous cell carcinoma; *HIST1H3B* (19%) and *MYCN* (13%) in liver cancer; and *HIST1H3B* (12%) in breast cancer. Among the highly recurrent oncogenes amplified with overexpression in specific tumors, including *MYC* in colorectal cancer, *MYCN* in colorectal and liver cancers, and *HIST1H3B* and *EZH2* in colorectal, lung, liver, and breast cancers, there was no significance between their status of amplification with overexpression and cancer stage (Supplementary Table 3) when significance was defined as *P* < 0.05 (Fisher’s exact test). However, *MYCN* amplification with overexpression was observed in colorectal cancer samples more frequently with stage III and IV than with stage I and II (*P* = 0.0672). In GISTs, 57% of samples exhibited *KIT* amplification-dependent overexpression. Another notable finding was that 21 of 25 samples (84%) with *FOXL2* amplification-dependent overexpression were derived from squamous cell carcinoma of the lung (10 of 56), head and neck (9 of 101), and esophagus (2 of 14), and its frequency was 12% of a total of 176 squamous cell carcinoma samples (Supplementary Fig. 3).

**Table 2:** Frequencies of overexpression with amplification of 64 oncogenes in individual cancer types.

Gene	Oncogene type	CRC	Lg-ad	Lg-sq	St	HN	Liv	Bre	Pan	Sar	Ute	Kid	EGJ	Eso	GIST	Ov	Mel	All
*MYC*	Amplification-based	25	1	2	3	1	3	1	0	5	0	6	6	0	0	0	0	10
*SKP2*	Amplification-based	0	3	21	5	8	1	0	0	5	0	0	25	7	0	0	0	3
*MYCN*	Amplification-based	2	3	9	3	0	13	4	0	0	5	0	6	0	0	0	0	3
*NKX2-1*	Amplification-based	2	2	7	1	7	0	1	3	0	0	0	13	36	0	0	0	2
*MDM2*	Amplification-based	0	3	0	1	1	0	2	0	16	0	0	0	0	7	0	17	1
*MYCL v.1/2*	Amplification-based	1	2	5	1	0	0	2	0	0	0	0	0	7	0	7	0	1
*MET*	Mutation-based	18	7	0	1	9	0	0	3	0	0	12	6	14	0	7	17	9
*HIST1H3B*	Mutation-based	4	8	7	4	4	19	12	0	11	0	0	19	7	0	14	17	7
*EZH2*	Mutation-based	6	6	5	2	3	7	6	3	5	5	0	13	7	0	14	0	6
*EGFR v.3*	Mutation-based	2	6	16	1	7	1	1	6	0	0	0	0	21	0	0	0	3
*EGFR v.1*	Mutation-based	0	8	14	1	7	0	1	3	0	0	12	0	14	0	0	0	3
*CARD11*	Mutation-based	6	1	2	1	2	2	0	0	0	0	0	6	7	0	7	8	3
*ERBB2*	Mutation-based	2	2	0	3	2	0	8	0	0	5	0	19	0	0	0	0	2
*FOXL2*	Mutation-based	0	0	18	1	9	0	0	0	0	0	0	0	14	0	0	0	2
*ALK*	Mutation-based	0	3	9	1	0	2	1	3	5	5	0	0	0	0	0	0	2
*KIT*	Mutation-based	0	1	0	0	0	3	0	0	0	0	12	0	0	57	0	17	1
*FGFR3*	Mutation-based	1	1	5	1	0	1	1	0	5	5	0	0	0	0	0	0	1
*DNMT3A v.1–3*	Mutation-based	0	1	2	1	1	1	1	0	5	5	0	0	0	0	0	0	1
*RET v.2*	Mutation-based	0	2	2	1	3	0	1	0	0	0	0	0	0	0	0	0	1
*FGFR2*	Mutation-based	0	0	0	4	1	0	0	0	0	5	0	0	0	0	0	0	1

Frequencies were calculated by determining the number of samples overexpressed and amplified (combining high and moderate amplification) and presented as percentages. Genes overexpressed and amplified in ≥10 samples are listed. Types of tumors were as follows: CRC, colorectal cancer; Lg-ad, lung adenocarcinoma; Lg-sq, lung squamous cell carcinoma; St, stomach cancer; HN, head and neck cancer; Liv, liver cancer; Bre, breast cancer; Pan, pancreatic cancer; Sar, sarcoma; Ute, uterine cancer; Kid, kidney cancer; EGJ, esophagogastric junction cancer; Eso, esophageal cancer; GIST, gastrointestinal stromal tumor; Ov, ovarian cancer; and Mel, melanoma. Frequencies observed in all 1,454 tumor samples are indicated as “All”.

**Figure 3:**
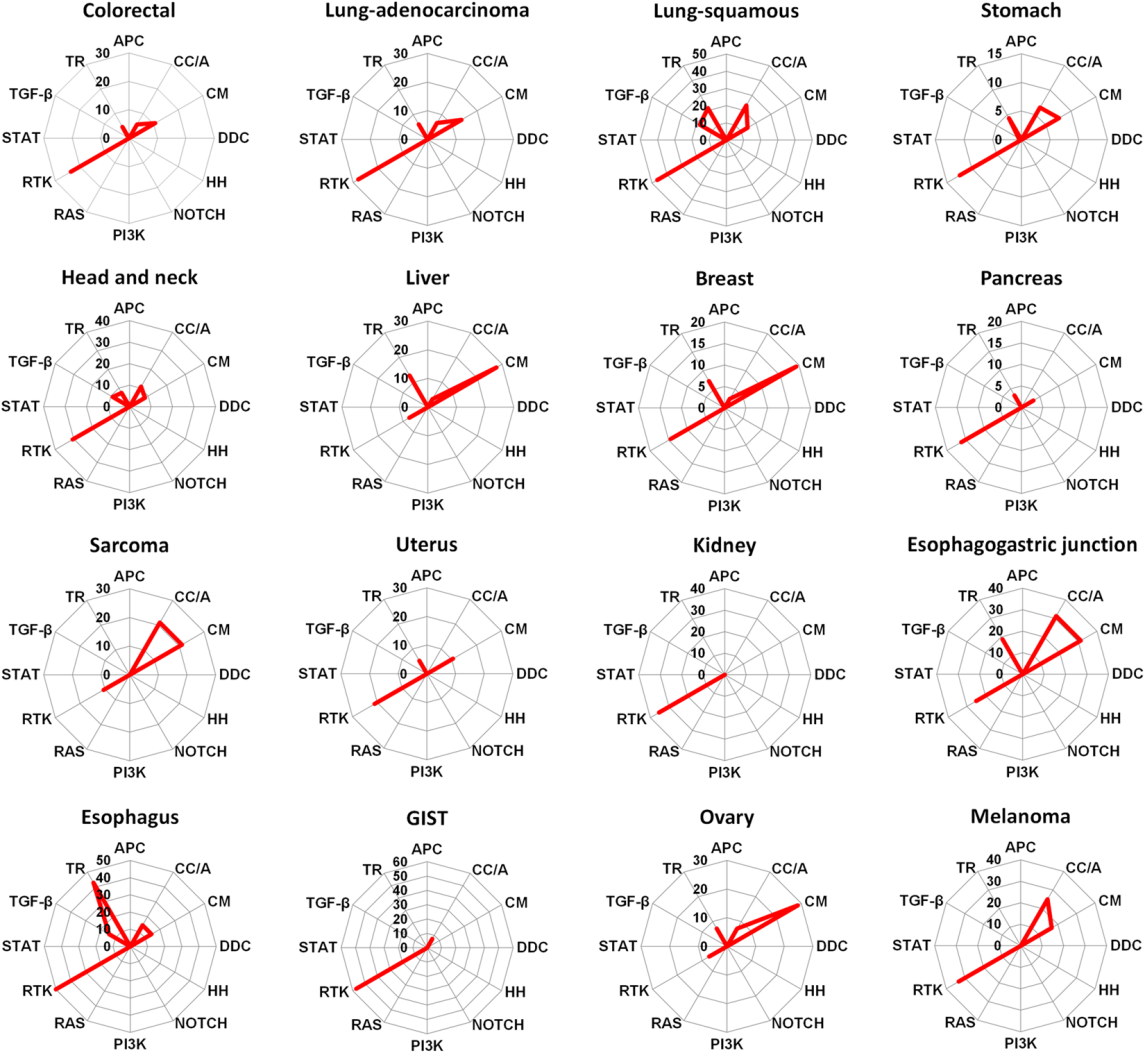
Radar charts comparing the 12 core signaling pathways involved in amplified and overexpressed oncogenes in individual cancer types. Each axis of the diagrams represents a percentage of frequencies of pathway activation derived from amplified and overexpressed oncogenes, as listed in Table 2. As listed in Supplementary Table 1, 138 driver genes were assigned to the 12 signaling pathways, including APC, cell cycle/apoptosis (CC/A), chromatin modification (CM), DNA damage control (DDC), Hedgehog (HH), NOTCH, PI3K, RAS, receptor tyrosine kinase (RTK), STAT, TGF-β, and transcriptional regulation (TR).

Additionally, we classified 138 driver genes into the 12 core signaling pathways (Supplementary Table 1). There were five pathways, including cell cycle/apoptosis (CC/A), chromatin modification (CM), receptor tyrosine kinases (RTK), TGF-β, and transcriptional regulation (TR), related to the amplified and overexpressed oncogenes shown in Table 2. As shown in Fig. 3, the activation of receptor tyrosine kinases pathway was involved in all types of tumors, whereas the CM pathway was activated in the samples from liver and ovarian cancers, and sarcoma. Furthermore, the activation patterns were similar for samples from lung squamous cell carcinoma, head and neck, and esophageal cancers. Since amplification-dependent overexpression is predicted to be involved in oncogenesis, the genes amplified with overexpression are potential target molecules for anti-cancer agents.

### Cancer-related genes with potential oncogenic driver gene amplification

**Figure 4:**
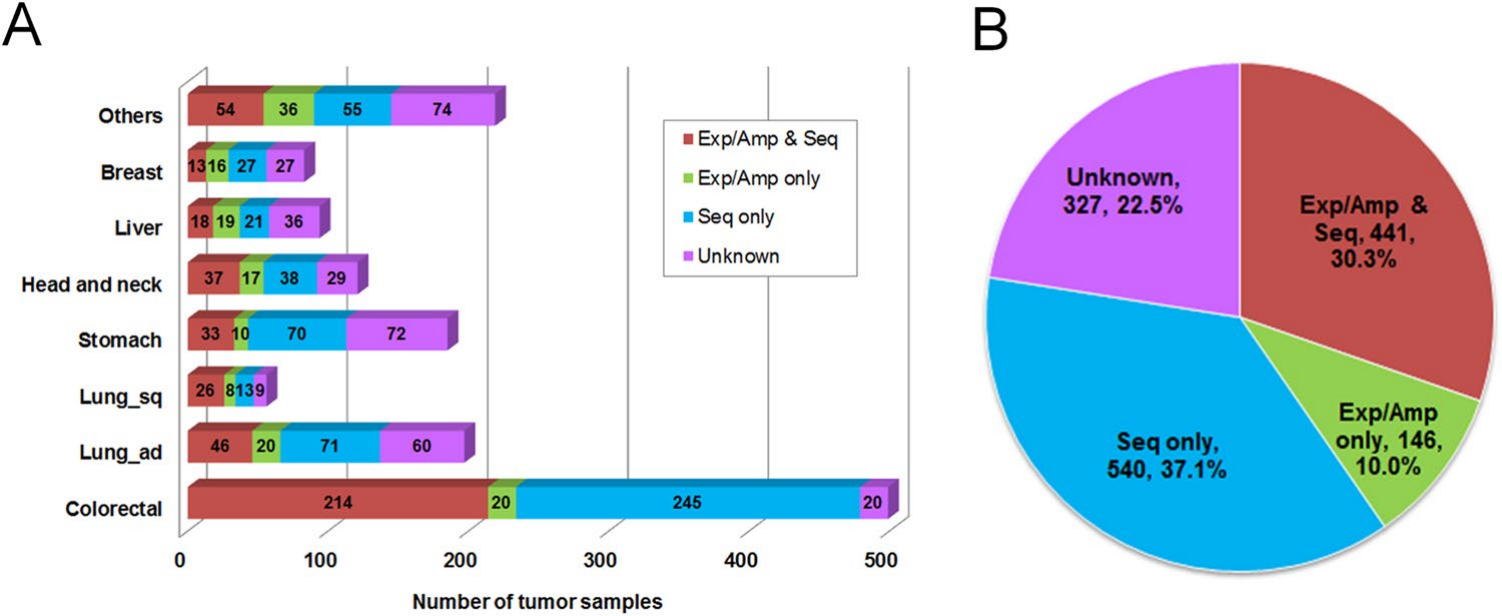
Genomic aberrations of cancer driver genes in 1,454 solid tumors. (**A**) Bar graph depicting the number of samples having somatic structural alterations in individual tumor types. Genomic alterations were grouped by cases of overexpression with gene amplification in 64 oncogenes (Exp/Amp), cases of mutations, insertions, and deletions in 138 driver genes, along with the presence of fusion genes (Seq), and cases without any alterations (Unknown). (**B**) Pie chart depicting the frequency of somatic structural alterations in all tumor samples.

**Figure 5:**
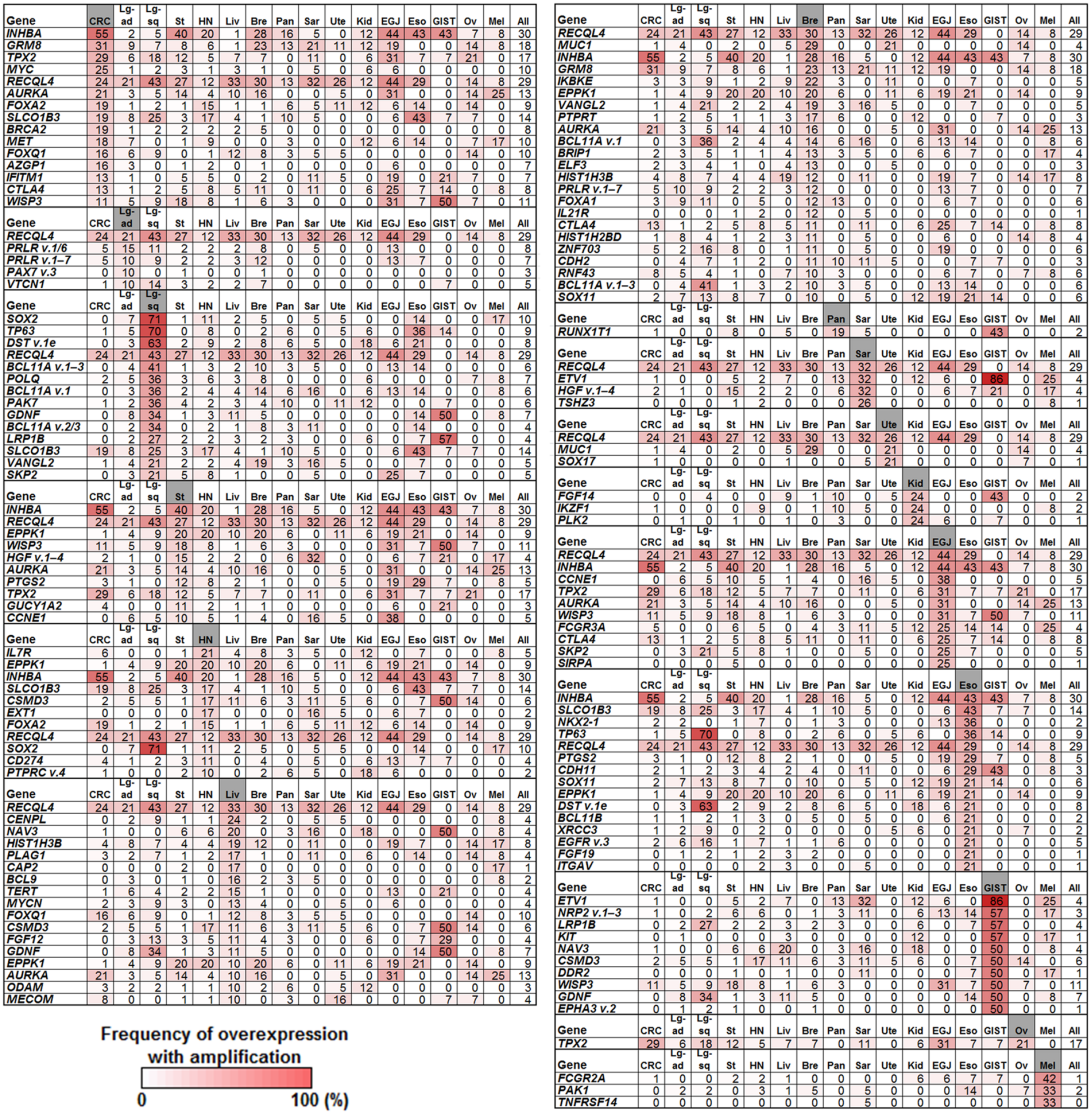
Frequency of overexpression with gene amplification of 820 cancer-related genes in individual tumor types. Genes overexpressed with ≥5-fold and a genomic copy number of ≥3 are ordered by type of tumor, as shaded. In colorectal (CRC), squamous cell carcinoma of the lung (Lg-sq), stomach (St), head and neck (HN), liver (Liv), and breast (Bre) cancer, genes with ≥10% frequency are shown. For the remaining tumor types, including tumors from lung adenocarcinoma (Lg-ad), pancreas (Pan), sarcoma (Sar), uterus (Ute), kidney (Kid), esophagogastric junction (EGJ), esophagus (Eso), gastrointestinal stromal tumor (GIST), ovary (Ov), and melanoma (Mel), genes with frequencies approximately ≥20% are listed. Frequencies observed in all 1,454 tumor samples are indicated as “All”. The frequency is indicated as a heat map (range 0–100%).

**Figure 6:**
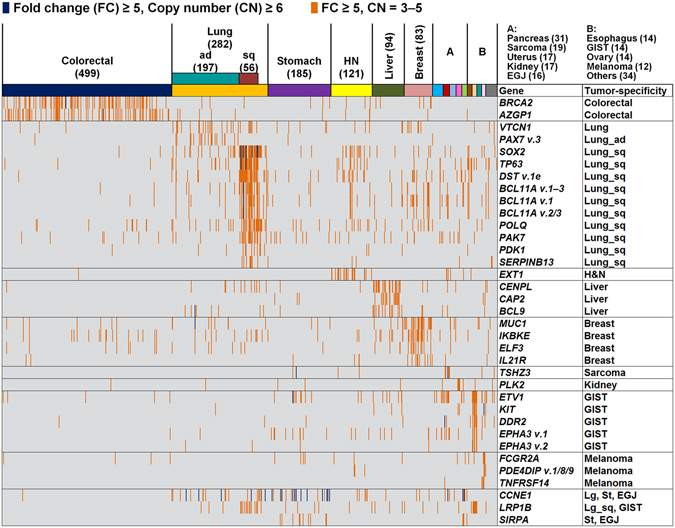
Tumor-specific overexpression with gene amplification among 820 cancer-related genes in 1,454 solid tumors. Genes overexpressed ≥5-fold with high (copy number ≥6) and moderate (copy number 3–5) genomic amplification are indicated by dark blue and orange bars, respectively. The tumor specificity of each gene is indicated on the right. As indicated at the top, samples are arranged by tumor type, including tumors from colorectal, lung, stomach, head and neck (HN), liver, breast, pancreas, sarcoma, uterus, kidney, esophagogastric junction (EGJ), esophagus, gastrointestinal stromal tumor (GIST), ovary, melanoma, and other types of tumors. The number of samples of each type of tumor is indicated in parentheses.

Besides CNVs, we analyzed sequence-based alterations, including substitutions, insertions, and deletions in the 138 driver genes, along with 491 fusion genes. Integration of sequence- and amplification-based alteration data revealed that genomic driver aberrations were present in 1,127 of 1,454 tumors (78%) (Fig. 4, Supplementary Fig. 4), while the driver origins of the remaining 327 tumors (22%) were undetermined. Next, we expanded the number of target genes to 820 to clarify whether any other oncogenic driver gene amplifications were involved in the 327 tumors undetermined driver alterations by the initial analyses. Referring to various databases and publications^19–27^ (https://www.bcm.edu/research/medical-genetics-labs/test_detail.cfm?testcode=9705), we selected 820 cancer-related genes, referred to as SCC-820 (Supplementary Table 2). The SCC-820 set of genes includes the 138 cancer driver genes^1^. Initially, the levels of expression and copy numbers of SCC-820 genes were characterized in 1,454 tumors, and 589 of 879 microarray probes corresponding to SCC-820 genes exhibited amplification-dependent overexpression in 1,251 tumors (86%). Among them, *INHBA* and *RECQL4* were frequently overexpressed and amplified in various tumor types (Fig. 5). *INHBA* overexpression promotes cell proliferation in esophageal adenocarcinoma^28^, while *RECQL4* is associated with breast cancer tumor aggressiveness, due to both amplification and overexpression^29^. Thus, these genes have been potentially associated with oncogenesis in previous reports. In addition, *SOX2* and *TP63*, map to chromosome 3q26.32–q29, were frequently overexpressed and amplified specifically in lung squamous cell carcinoma tumors (Fig. 6 and Supplementary Fig. 5A). Overexpression of *CCNE1* with high-level genomic amplification was identified in lung, stomach, sarcoma, and esophagogastric junction tumors. Genes co-localizing with *CCNE1* on chromosome 19q12–13.12, including *TSHZ3*, *CEBPA*, *PDCD2L*, *ALKBH6*, and *KMT2B*, were also co-overexpressed and co-amplified in some tumors.

*SOX2* ^30^ and *CCNE1* ^31^ have previously been reported to function as driver oncogenes. These analyses of SCC820 genes identified additional genes (relative to those identified by analysis of the data on 64 oncogenes) with oncogenic potential that exhibited amplification-dependent overexpression.

### Potential oncogenic driver gene amplification in tumors in which driver genes were not initially identified

Among the 1,454 tumors, 327 were categorized as having undetermined driver origins following mutation, copy number, and expression analyses of 138 driver and 491 fusion genes (Fig. 4 and Supplementary Fig. 4). For these 327 tumors, overexpression and amplification analysis using the SCC820 genes identified 214 (65%) and 113 (35%) tumors with and without amplification-dependent overexpression, respectively. There were 16 genes identified only in the 327 tumors (Table 3). Since no genomic alterations, other than amplification-dependent overexpression, were identified, these genes are candidate driver genes. Recurrent amplification-dependent overexpression of *AXL*, which encodes a receptor tyrosine kinase, was identified in two sarcoma samples. Among the 16 genes, *AXL* ^32^ and *DIS3* ^33^ have been previously reported to be associated with oncogenesis.

**Table 3:** Identification of potential oncogenic driver genes with amplification in tumors in which driver genes were not identified by initial analyses.

Gene	Copy number	Fold change (mRNA)	Locus	Tumor	Description	Observation on oncogenesis by amplification-dependent overexpression	Reference	Co-overexpressed and co-amplified genes in this study
*AXIN1*	3	8.06	16p13.3	Stomach	Axin 1			
*AXL*	3	12.91	19q13.2	Sarcoma	AXL receptor tyrosine kinase	Promoting proliferation and tumorigenicity	42	
	3	11.29		Sarcoma^1^				
*CD70*	3	5.47	19p13.3	Breast	CD70 molecule			
*CNKSR1*	3	23.29	1p36.11	Liver	Connector enhancer of kinase suppressor of Ras 1			
*DIS3*	3	5.93	13q21.33	Liver^2^	DIS3 exosome endoribonuclease and 3′–5′ exoribonuclease	Promoting proliferation and invasiveness	33	
*DST*	3	7.56	6p12.1	Pancreas	Dystonin			
*EZH1*	4	6.60	17q21.2	Liver^2^	Enhancer of zeste 1 polycomb repressive complex 2 subunit			BRCA1 and ETV4 on chromosome 17q21.2–q21.31
*FAM175A*	3	6.37	4q21.23	Kidney^3^	Family with sequence similarity 175, member A			
*FLCN*	4	7.74	17p11.2	Sarcoma^1^	Folliculin			
*FOXO1*	3	7.02	13q14.11	Kidney^3^	Forkhead box O1			
*HAUS3*	3	5.12	4p16.3	Kidney^3^	HAUS augmin-like complex, subunit 3			
*MTR*	3	6.33	1q43	Liver	5-Methyltetrahydrofolate-homocysteine methyltransferase			
*PER1*	3	7.60	17p13.1	GIST	Period circadian clock 1			
*PIK3CB*	5	5.67	3q22.3	Uterus	Phosphatidylinositol-4,5-bisphosphate 3-kinase, catalytic subunit beta			
*TSC2*	3	5.76	16p13.3	Breast^4^	Tuberous sclerosis 2			
*ZNF384*	10	5.24	12p13.31	Breast^4^	Zinc finger protein 384			ETV6 and RAD52 on chromosome 12p13.33–p13.31

^1^ *AXL* and *FLCN* data were obtained from the same patient with sarcoma.

^2^ *DIS3* and *EZH1* data were obtained from the same patient with liver cancer.

^3^ *FAM175A*, *FOXO1*, and *HAUS3* data were obtained from the same patient with kidney cancer.

^4^ *TSC2* and *ZNF384* data were obtained from the same patient with breast cancer.

## Discussion

We conducted integrative GEP and CNV analyses of 1,454 tumors to identify potential driver genes with amplification-dependent overexpression. When the number of copies of a gene increases, its expression is predicted to be elevated; however, there has been a report of gene amplification and copy number gain in the absence of overexpression^6^. Therefore, for the identification of driver genes, it is essential to investigate gene expression levels alongside copy number analysis. In this study, we also observed discrepancies between genomic copy number increase and levels of gene expression, particularly in cases where copy numbers were moderately elevated (3–5 copies) (Fig. 2). Moreover, elevation of gene expression was not always accompanied by copy number gain (Fig. 2 and Table 1).

Although there are no clear criteria defining gene amplification according to the number of copies, arbitrary numbers have been provided for some genes. For *ALK*, the presence of ≥6 copies has been defined as amplification, while having 3–5 copies was defined as copy number gain^34^. For *RET*, amplification defined as the presence of ≥5 copies, with copy number gain set at 3–4 copies^35^. Similar to these examples, we defined our own criteria for the grade of amplification by copy number as follows; copy numbers of ≥6 were defined as high-level amplification, while copy numbers of 3–5 were considered as moderate amplification. Depending on the number of copies, particular amplified sequences have been linked to clinical features, including *MYCN* and poor prognosis, and *ERBB2* and drug sensitivity^6^. In addition, *SKP2* overexpression and amplification has been related to metastasis in lung squamous cell carcinoma^36^. It remains to be seen whether the overexpressed and amplified genes identified in this study demonstrate such clinical significance. In particular, it will be of interest to determine differences between the two groups classified as having high and moderate amplification of *ERBB2* and *EGFR* (Fig. 2B).

Vogelstein *et al.* selected 138 driver genes consisting of 64 oncogenes and 74 tumor suppressor genes (TSGs), the former of which were classified into two groups, including 10 amplification-based and 54 mutation-based oncogenes. Among the 10 amplification-based oncogenes, we identified that six genes (*MDM2*, *MYC*, *MYCL*, *MYCN*, *NKX2-1*, and *SKP2*) were amplified and overexpressed in ≥10 tumor samples (Supplementary Fig. 3). As among the most frequently amplified oncogenes^37–39^, *MYC* was amplified and overexpressed in a wide range of tumor tissues. Amplification with overexpression of *MYCL*, *NKX2-1*, and *SKP2* in lung cancer, and *MDM2* in sarcoma was observed as previously reported^6^. Although *MYCN* amplification with overexpression was observed in neuroblastoma, sarcoma, and lung cancer, our study identified that *MYCN* was amplified and overexpressed in 13% of liver cancer samples. Among the remaining four genes (*CCND1*, *LMO1*, *MDM4*, and *NCOA3*), we observed no amplification or overexpression of *MDM4*, which is amplified and overexpressed in glioma and retinoblastoma^6^, in our analysis of 1,454 tumors. Amplification with overexpression of *CCND1* and *NCOA3*, which was previously observed in breast cancer^6^, were detected in four samples from colorectal, head and neck, liver, and ovarian cancer for *CCND1* and eight samples from three sarcoma, two esophageal cancer, and one sample each from colorectal, lung, and breast cancer for *NCOA3*. Although *LMO1* duplication was associated with more advanced disease and survival in neuroblastoma^40^, our analysis identified four samples from lung adenocarcinoma exhibiting *LMO1* amplification and overexpression, three of which were derived from stage I and no stage information provided for the remaining sample.

The analysis of 64 known driver oncogenes revealed amplification-dependent overexpression in 587 of 1,454 tumors (40%) (Fig. 4B). The subsequent extended analysis including 820 cancer-related genes narrowed down the candidate driver genes associated with amplification-dependent overexpression, particularly in tumors with unidentified driver origins after the analyses of the 138-driver and 491-fusion genes. Sixteen genes (*AXIN1*, *AXL*, *CD70*, *CNKSR1*, *DIS3*, *DST*, *EZH1*, *FAM175A*, *FLCN*, *FOXO1*, *HAU3*, *MTR*, *PER1*, *PIK3CB*, *TSC2*, and *ZNF384*) were identified only in the 327 tumors that had undetermined drivers after the initial analyses. Of these, *AXL* and *DIS3* have previously been suggested to have oncogenic functions^32, 33^. Recurrent amplification-dependent overexpression of *AXL* was observed only in two sarcomas (myxofibrosarcoma and leiomyosarcoma). In addition, a liposarcoma tumor sample among the 1,127 tumors with identified driver origins after the initial analyses exhibited a fold change in *AXL* expression approaching the cutoff level (FC = 4.97) and had four genomic copies of this gene. Including this case, *AXL* amplification-dependent overexpression was observed in three of a total of 19 sarcoma samples (16%). *AXL* (previously known as *UFO*), is a member of the TMA (*TYRO3*, *MER*, and *AXL*) receptor tyrosine kinase family and has important roles in various cancer processes [reviewed in ref. 41]. *AXL* amplification has been identified in colorectal cancer^42^ Moreover, *AXL* overexpression has been observed in many solid and hematopoietic malignancies, including Ewing sarcoma tumor tissues^43^ and sarcoma cell lines^44^. Since the relationship between levels of amplification and overexpression is currently unclear, from a clinical perspective, it will be of interest to determine the prognostic significance of *AXL* amplification-dependent overexpression in sarcoma tumors in the future.

Searching for genes overexpressed as a result of genomic amplification is one method of driver gene identification. It will be necessary and appropriate to eliminate passenger genes from these candidate drivers, particularly those derived from the analysis of 820 cancer-related genes using sophisticated *in vitro* and *in silico* experiments^7^. Another future challenge is to identify driver genes in the 113 tumors where the driver origin remained unknown after analysis on the SCC-820 dataset. Since our analyses were conducted in-house, the data we obtained from the multi-omics project can readily be coupled with clinical data to provide therapeutic and prognostic information for individual patients, as a step towards the development of personalized medicine.

## Methods

### Study setting

Ethical approval for all experimental protocols and study was obtained from the institutional review board at the Shizuoka Cancer Center (Authorization Number: 25–33). Written informed consent was obtained from all patients enrolled in the study. All experiments using clinical samples were carried out in accordance with the approved guidelines.

### Clinical samples

Tumor tissue samples with sizes corresponding to weights of ≥0.1 g were dissected from surgical specimens, along with samples of surrounding normal tissue. The areas from which tumor samples were dissected were visually assessed as containing ≥50% tumor content. For RNA analysis, tissue samples were submerged in RNAlater solution (Thermo Fisher Scientific), minced, and stored overnight at 4 °C before RNA extraction. For DNA analysis, tumor and normal tissues were immediately frozen in liquid nitrogen before DNA extraction. In addition, whole blood was collected as a control for whole exome sequencing.

### RNA isolation

Total RNA was extracted from approximately 10 mg of minced tissue samples using the miRNeasy Mini Kit (Qiagen) according to the manufacturer’s instructions. Initially, tissue samples were mixed with QIAzol reagent from the kit and then ground with a TissueLyzer II (Qiagen) using a 5-mm zirconia bead for 10 min at room temperature. RNA samples were quantified using a NanoDrop spectrophotometer (Thermo Fisher Scientific) and their quality was assessed using an Agilent 2100 Bioanalyzer (Agilent Technologies) with an RNA 6000 Nano total RNA Kit (Agilent Technologies).

### GEP analysis

RNA samples with RNA integrity number ≥6.0 was used for microarray analysis. Total RNA (100 ng) was amplified and fluorescence-labeled using the One-Color Low Input Quick Amp Labeling Kit (Agilent Technologies) according to the manufacturer’s instructions. Labeled samples were hybridized to the SurePrint G3 Human Gene Expression 8 × 60 K v2 Microarray (Agilent Technologies), which has 50,599 probes capable of detecting 29,833 genes registered in the Entrez Gene Database, published by the National Center for Biotechnology Information. Expression levels were calculated using previously described methods^45^, and data derived from tumor tissue samples collated with that from corresponding adjacent normal tissue specimens. Hybridization signals were detected using a DNA Microarray Scanner (Agilent Technologies) and scanned images were analyzed using Agilent Feature Extraction software. Microarray analysis was performed in accordance with the MIAME guidelines^46^.

Data analysis was performed using GeneSpring GX software (Agilent Technologies) and Microsoft Excel. Probes to be analyzed were selected according to the reference genome sequence, hg19, obtained from the UCSC Genome Browser^47^. Raw signal intensity values were log transformed and normalized to the 75th percentile. The fold change between tumor and normal tissues from the same patient was calculated using the normalized intensity values. Probes expressed at raw signal values < 10 in both tumor and normal tissues were excluded from further analysis. GEP data of SCC-820 genes are included in Supplementary Dataset 1 as MIAME-compliant data.

### DNA isolation and WES analysis

DNA was extracted from tissue and blood samples using a QIAamp Kit (Qiagen) according to the manufacturer’s instructions, and subjected to WES on the Ion Proton System (Thermo Fisher Scientific) as reported previously^48, 49^. For data analysis, single-nucleotide variants with quality scores <30 or depth of coverage <20 were discarded. Torrent Suite software (ver. 4.4) was used to convert raw binary data into sequence reads that were mapped to the reference human genome (UCSC, hg19). Somatic mutations were identified by comparing data from tumor and corresponding blood samples. Driver mutations in 138 known driver genes^1^ were defined as those identified as pathogenic in the ClinVar database^50^, or those contained in the Database of Curated Mutations (DoCM, http://docm.genome.wustl.edu) or the UMD TP53 mutation database^51^. In addition, nonsense, frameshift, and splice site mutations in TSGs among 138 driver genes were also classified as driver mutations.

CNVs were detected using Ion Reporter Software Copy Number Variation Analysis (Thermo Fisher Scientific). The CNV detection algorithm was based on a hidden Markov model. CNVs with confidence scores ≥10 were included in the analysis. Using this system, the maximum copy number calculated was 10. CNV data of SCC-820 genes are included in Supplementary Dataset 2. The WES data was applied to estimate tumor purity using an *in silico* method^52^, which is included in Supplementary Dataset 2.

### Detection of fusion genes

Fusion gene analysis was performed using the Ion Proton System, as previously reported^53^. In brief, total RNA (10 ng) was used as a template to prepare cDNA using the SuperScript VILO cDNA Synthesis Kit (Thermo Fisher Scientific). The Ion AmpliSeq Library Kit 2.0 (Thermo Fisher Scientific) and the Ion Proton Sequencing 200 Kit (Thermo Fisher Scientific) were used to construct an Ion Torrent adapter-ligated library and perform nucleotide sequencing, respectively, according to the manufacturer’s protocols. All data were analyzed using the Ion Reporter server. The Ion AmpliSeq RNA Fusion workflow (Thermo Fisher Scientific) was used to detect fusion transcripts from a panel of 491 fusion genes.

## Electronic supplementary material

Supplementary Information

Dataset 1_1

Dataset 1_2

Dataset 1_3

Dataset 2
