# Supplementary material for: Integrated analysis of gene expression and copy number identified potential cancer driver genes with amplification-dependent overexpression in 1,454 solid tumors
Source: Sci Rep. 2017 Apr 4;7:641. doi: 10.1038/s41598-017-00219-3 (PMC5428069; doi:10.1038/s41598-017-00219-3)
Supplement: Supplementary file 1 — Supplementary Information [file 41598_2017_219_MOESM1_ESM.pdf]

Supplementary Information

Integrated analysis of gene expression and copy number identified potential cancer driver genes with amplification-dependent overexpression in 1,454 solid tumors

Keiichi Ohshima[1*], Keiichi Hatakeyama[1], Takeshi Nagashima[2,3], Yuko Watanabe[1], Kaori Kanto[1], Yuki Doi[1], Tomomi Ide[1], Yuji Shimoda[2,3], Tomoe Tanabe[2,3], Sumiko Ohnami[2], Shumpei Ohnami[2], Masakuni Serizawa[4], Koji Maruyama[5], Yasuto Akiyama[6], Kenichi Urakami[2], Masatoshi Kusuhara[4,7], Tohru Mochizuki[1] & Ken Yamaguchi[8]

[1]Medical Genetics Division, [2]Cancer Diagnostics Research Division, [4]Drug Discovery and Development Division, [5]Experimental Animal Facility, [6]Immunotherapy Division, [7]Region Resources Division, Shizuoka Cancer Center Research Institute, Shizuoka 411-8777, Japan. [3]SRL, Inc., Tokyo 163-0409, Japan. [8]Shizuoka Cancer Center Hospital and Research Institute, Shizuoka 411-8777, Japan.

*Correspondence and requests for materials should be addressed to K.O. (email: k.ohshima@scchr.jp)

Table S1. List of 138 cancer driver genes.

Gene symbol	Classification	Type	Core pathway
ABL1	Oncogene	Mutation-based	Cell Cycle/Apoptosis
AKT1	Oncogene	Mutation-based	PI3K
ALK	Oncogene	Mutation-based	RTK*
AR	Oncogene	Mutation-based	Transcriptional Regulation
BCL2	Oncogene	Mutation-based	Cell Cycle/Apoptosis
BRAF	Oncogene	Mutation-based	RAS
CARD11	Oncogene	Mutation-based	Cell Cycle/Apoptosis
CBL	Oncogene	Mutation-based	RTK
CRLF2	Oncogene	Mutation-based	STAT
CSF1R	Oncogene	Mutation-based	RTK
CTNNB1	Oncogene	Mutation-based	APC
DNMT1	Oncogene	Mutation-based	Chromatin Modification
DNMT3A	Oncogene	Mutation-based	Chromatin Modification
EGFR	Oncogene	Mutation-based	RTK
ERBB2	Oncogene	Mutation-based	RTK
EZH2	Oncogene	Mutation-based	Chromatin Modification
FGFR2	Oncogene	Mutation-based	RTK
FGFR3	Oncogene	Mutation-based	RTK
FLT3	Oncogene	Mutation-based	RTK
FOXL2	Oncogene	Mutation-based	TGF-β
GATA2	Oncogene	Mutation-based	Transcriptional Regulation
GNA11	Oncogene	Mutation-based	RAS
GNAQ	Oncogene	Mutation-based	RAS
GNAS	Oncogene	Mutation-based	RAS
H3F3A	Oncogene	Mutation-based	Chromatin Modification
HIST1H3B	Oncogene	Mutation-based	Chromatin Modification
HRAS	Oncogene	Mutation-based	RAS
IDH1	Oncogene	Mutation-based	Chromatin Modification
IDH2	Oncogene	Mutation-based	Chromatin Modification
JAK1	Oncogene	Mutation-based	STAT
JAK2	Oncogene	Mutation-based	STAT
JAK3	Oncogene	Mutation-based	STAT

KIT	Oncogene	Mutation-based	RTK
KLF4	Oncogene	Mutation-based	Transcriptional Regulation
KRAS	Oncogene	Mutation-based	RAS
MAP2K1	Oncogene	Mutation-based	RAS
MED12	Oncogene	Mutation-based	Cell Cycle/Apoptosis
MET	Oncogene	Mutation-based	RTK
MPL	Oncogene	Mutation-based	STAT
MYD88	Oncogene	Mutation-based	Cell Cycle/Apoptosis
NFE2L2	Oncogene	Mutation-based	Cell Cycle/Apoptosis
NRAS	Oncogene	Mutation-based	RAS
PDGFRA	Oncogene	Mutation-based	RTK
PIK3CA	Oncogene	Mutation-based	PI3K
PPP2R1A	Oncogene	Mutation-based	Cell Cycle/Apoptosis
PTPN11	Oncogene	Mutation-based	RAS
RET	Oncogene	Mutation-based	RTK
SETBP1	Oncogene	Mutation-based	Chromatin Modification
SF3B1	Oncogene	Mutation-based	Transcriptional Regulation
SMO	Oncogene	Mutation-based	Hedgehog
SPOP	Oncogene	Mutation-based	Chromatin Modification
SRSF2	Oncogene	Mutation-based	Transcriptional Regulation
TSHR	Oncogene	Mutation-based	Transcriptional Regulation
U2AF1	Oncogene	Mutation-based	Transcriptional Regulation
CCND1	Oncogene	Amplification-based	Cell Cycle/Apoptosis
LMO1	Oncogene	Amplification-based	Transcriptional Regulation
MDM2	Oncogene	Amplification-based	Cell Cycle/Apoptosis
MDM4	Oncogene	Amplification-based	Cell Cycle/Apoptosis
MYC	Oncogene	Amplification-based	Transcriptional Regulation
MYCL	Oncogene	Amplification-based	Transcriptional Regulation
MYCN	Oncogene	Amplification-based	Transcriptional Regulation
NCOA3	Oncogene	Amplification-based	Chromatin Modification
NKX2-1	Oncogene	Amplification-based	Transcriptional Regulation
SKP2	Oncogene	Amplification-based	Cell Cycle/Apoptosis
ACVR1B	TSG	Mutation-based	TGF-β
AMER1	TSG	Mutation-based	APC
APC	TSG	Mutation-based	APC

ARID1A	TSG	Mutation-based	Chromatin Modification
ARID1B	TSG	Mutation-based	Chromatin Modification
ARID2	TSG	Mutation-based	Chromatin Modification
ASXL1	TSG	Mutation-based	Chromatin Modification
ATM	TSG	Mutation-based	DNA Damage Control
ATRX	TSG	Mutation-based	Chromatin Modification
AXIN1	TSG	Mutation-based	APC
B2M	TSG	Mutation-based	RAS
BAP1	TSG	Mutation-based	DNA Damage Control
BCOR	TSG	Mutation-based	Transcriptional Regulation
BRCA1	TSG	Mutation-based	DNA Damage Control
BRCA2	TSG	Mutation-based	DNA Damage Control
CASP8	TSG	Mutation-based	Cell Cycle/Apoptosis
CDC73	TSG	Mutation-based	Cell Cycle/Apoptosis
CDH1	TSG	Mutation-based	APC
CDKN2A	TSG	Mutation-based	Cell Cycle/Apoptosis
CEBPA	TSG	Mutation-based	Transcriptional Regulation
CIC	TSG	Mutation-based	RAS
CREBBP	TSG	Mutation-based	Chromatin Modification
CYLD	TSG	Mutation-based	Cell Cycle/Apoptosis
DAXX	TSG	Mutation-based	Chromatin Modification
EP300	TSG	Mutation-based	Chromatin Modification
FBXW7	TSG	Mutation-based	NOTCH
FUBP1	TSG	Mutation-based	Cell Cycle/Apoptosis
GATA1	TSG	Mutation-based	Transcriptional Regulation
GATA3	TSG	Mutation-based	Transcriptional Regulation
HNF1A	TSG	Mutation-based	APC
KDM5C	TSG	Mutation-based	Chromatin Modification
KDM6A	TSG	Mutation-based	Chromatin Modification
KMT2C	TSG	Mutation-based	Chromatin Modification
KMT2D	TSG	Mutation-based	Chromatin Modification
MAP3K1	TSG	Mutation-based	RAS
MEN1	TSG	Mutation-based	Chromatin Modification
MLH1	TSG	Mutation-based	DNA Damage Control
MSH2	TSG	Mutation-based	DNA Damage Control

MSH6	TSG	Mutation-based	DNA Damage Control
NCOR1	TSG	Mutation-based	Chromatin Modification
NF1	TSG	Mutation-based	RAS
NF2	TSG	Mutation-based	APC
NOTCH1	TSG	Mutation-based	NOTCH
NOTCH2	TSG	Mutation-based	NOTCH
NPM1	TSG	Mutation-based	Cell Cycle/Apoptosis
PAX5	TSG	Mutation-based	Chromatin Modification
PBRM1	TSG	Mutation-based	Chromatin Modification
PHF6	TSG	Mutation-based	Transcriptional Regulation
PIK3R1	TSG	Mutation-based	PI3K
PRDM1	TSG	Mutation-based	Chromatin Modification
PTCH1	TSG	Mutation-based	Hedgehog
PTEN	TSG	Mutation-based	PI3K
RB1	TSG	Mutation-based	Cell Cycle/Apoptosis
RNF43	TSG	Mutation-based	APC
RUNX1	TSG	Mutation-based	Transcriptional Regulation
SETD2	TSG	Mutation-based	Chromatin Modification
SMAD2	TSG	Mutation-based	TGF-β
SMAD4	TSG	Mutation-based	TGF-β
SMARCA4	TSG	Mutation-based	Chromatin Modification
SMARCB1	TSG	Mutation-based	Chromatin Modification
SOCS1	TSG	Mutation-based	STAT
SOX9	TSG	Mutation-based	APC
STAG2	TSG	Mutation-based	DNA Damage Control
STK11	TSG	Mutation-based	PI3K
TET2	TSG	Mutation-based	Chromatin Modification
TNFAIP3	TSG	Mutation-based	Cell Cycle/Apoptosis
TP53	TSG	Mutation-based	Cell Cycle/Apoptosis
TRAF7	TSG	Mutation-based	Cell Cycle/Apoptosis
TSC1	TSG	Mutation-based	PI3K
VHL	TSG	Mutation-based	PI3K
WT1	TSG	Mutation-based	Chromatin Modification
CDKN2C	TSG	Deletion-based	Cell Cycle/Apoptosis
IKZF1	TSG	Deletion-based	Transcriptional Regulation

MAP2K4	TSG	Deletion-based	RAS

*RTK: receptor tyrosine kinase

Table S2. Panel of 820 cancer-related genes (SCC-820).

ABCB1	ABCC2	ABCC4	ABCG2	ABL1	ABL2	ACO1	ACVR1B	ACVR2A	ACVR2B
ADAMTS20	ADGRA2	ADGRB3	ADGRL3	ADNP	AFF1	AFF3	AJUBA	AKAP9	AKT1
AKT2	AKT3	ALK	ALKBH6	ALOX12B	ALPK2	AMER1	APC	APCDD1	APOL2
AR	ARAF	ARFRP1	ARHGAP35	ARID1A	ARID1B	ARID2	ARID5B	ARL11	ARNT
ASXL1	ASXL2	ASXL3	ATF1	ATM	ATP1A1	ATP5B	ATR	ATRX	AURKA
AURKB	AURKC	AXIN1	AXIN2	AXL	AZGP1	B2M	B4GALT3	BACH1	BAK1
BAP1	BARD1	BBC3	BCL10	BCL11A	BCL11B	BCL2	BCL2L1	BCL2L11	BCL2L2
BCL3	BCL6	BCL9	BCLAF1	BCOR	BCORL1	BCR	BIRC2	BIRC3	BIRC5
BLM	BLNK	BMPR1A	BRAF	BRCA1	BRCA2	BRD3	BRD4	BRE	BRIP1
BRWD3	BTG1	BTK	BUB1B	C11orf30	CACNA1D	CALR	CAP2	CARD11	CASC5
CASP8	CBFB	CBL	CBLB	CBLC	CCND1	CCND2	CCND3	CCNE1	CD1D
CD274	CD276	CD70	CD79A	CD79B	CDC27	CDC73	CDH1	CDH11	CDH12
CDH18	CDH2	CDH20	CDH5	CDK12	CDK4	CDK6	CDK8	CDKN1A	CDKN1B
CDKN2A	CDKN2B	CDKN2C	CEBPA	CENPL	CEP76	CERS2	CHD4	CHD8	CHEK1
CHEK2	CHUK	CIC	CKS1B	CMPK1	CNBD1	CNKSR1	CNOT3	COL1A1	COMT
CRBN	CREB1	CREBBP	CRIPAK	CRKL	CRLF2	CRTC1	CSF1R	CSF3R	CSMD3
CTCF	CTLA4	CTNNA1	CTNNB1	CTTN	CUL3	CUL4A	CUL4B	CUX1	CYLD
CYP17A1	CYP1B1	CYP2C19	CYP2C8	CYP2D6	CYP3A4	CYP3A5	DAXX	DCAF6	DCC
DCUN1D1	DDB1	DDB2	DDIT3	DDR1	DDR2	DDX11	DDX3X	DDX5	DEK
DIAPH1	DICER1	DIDO1	DIS3	DLC1	DNER	DNM2	DNMT1	DNMT3A	DNMT3B
DOT1L	DPYD	DST	E2F3	ECSCR	ECT2L	EED	EGFL7	EGFR	EGR3

EIF1AX	EIF2S2	EIF3A	EIF4A2	ELF3	EME2	EML4	EP300	EP400	EPCAM
EPHA2	EPHA3	EPHA5	EPHA7	EPHB1	EPHB2	EPHB4	EPHB6	EPPK1	ERBB2
ERBB3	ERBB4	ERCC1	ERCC2	ERCC3	ERCC4	ERCC5	ERG	ESR1	ESR2
ETS1	ETV1	ETV4	ETV5	ETV6	EWSR1	EXT1	EXT2	EZH1	EZH2
EZR	FAM129B	FAM175A	FAM46C	FANCA	FANCB	FANCC	FANCD2	FANCE	FANCF
FANCG	FANCI	FANCL	FANCM	FAS	FAT1	FAT3	FBXO11	FBXW7	FCGR1A
FCGR2A	FCGR3A	FGF10	FGF12	FGF14	FGF19	FGF23	FGF3	FGF4	FGF6
FGF7	FGFBP1	FGFR1	FGFR2	FGFR3	FGFR4	FH	FHIT	FKBP9	FLCN
FLI1	FLT1	FLT3	FLT4	FN1	FOXA1	FOXA2	FOXL2	FOXO1	FOXO3
FOXP1	FOXP4	FOXQ1	FUBP1	FZD1	FZR1	G6PD	GAB2	GATA1	GATA2
GATA3	GDNF	GID4	GNA11	GNA13	GNAQ	GNAS	GNB1	GPC3	GPS2
GREM1	GRIN2A	GRM3	GRM8	GSK3B	GSTP1	GUCY1A2	H3F3A	H3F3B	H3F3C
HAUS3	HCAR1	HDAC4	HGF	HIF1A	HIST1H1C	HIST1H1E	HIST1H2BD	HIST1H3B	HIST1H4E
HLA-A	HLA-B	HLA-G	HLF	HNF1A	HOOK3	HRAS	HSP90AA1	HSP90AB1	ICK
ICOSLG	IDH1	IDH2	IFITM1	IFITM3	IFNGR1	IGF1	IGF1R	IGF2	IGF2R
IKBKB	IKBKE	IKZF1	IL10	IL2	IL21R	IL6ST	IL7R	ING1	ING4
INHA	INHBA	INPP4A	INPP4B	INPPL1	INSR	IPO7	IRF4	IRS1	IRS2
ITGA10	ITGA9	ITGAV	ITGB2	ITGB3	ITPA	JAK1	JAK2	JAK3	JUN
KAT6A	KAT6B	KCNJ5	KDM5A	KDM5C	KDM6A	KDR	KEAP1	KIF5B	KIT
KLF4	KLF6	KLHL6	KMT2A	KMT2B	KMT2C	KMT2D	KNSTRN	KRAS	LAMP1
LATS1	LATS2	LCK	LIFR	LMO1	LPP	LRP1B	LRP2	LRRK2	LTF
LTK	MAF	MAFB	MAGEA1	MAGI1	MAGOH	MALAT1	MALT1	MAML2	MAN1B1

MAP2K1	MAP2K2	MAP2K4	MAP3K1	MAP3K13	MAP3K15	MAP3K7	MAP4K1	MAP4K3	MAPK1
MAPK8	MAPK8IP1	MARK1	MARK4	MAX	MBD1	MC1R	MCL1	MDC1	MDM2
MDM4	MECOM	MED12	MED23	MEF2A	MEF2B	MEN1	MET	MGA	MIR142
MITF	MLH1	MLLT10	MMP2	MN1	MNDA	MORC4	MPL	MRE11A	MSH2
MSH6	MTHFR	MTOR	MTR	MTRR	MTUS2	MUC1	MUTYH	MXRA5	MYB
MYC	MYCL	MYCN	MYD88	MYH11	MYH9	MYLK	MYOD1	NAV3	NBN
NBPF1	NCOA1	NCOA2	NCOA3	NCOA4	NCOR1	NEIL1	NF1	NF2	NFE2L2
NFKB1	NFKB2	NFKBIA	NIN	NKX2-1	NKX3-1	NLRP1	NOTCH1	NOTCH2	NOTCH3
NOTCH4	NPM1	NQO1	NRAS	NRP2	NSD1	NT5C2	NTN4	NTRK1	NTRK2
NTRK3	NUMA1	NUP214	NUP93	NUP98	NUTM1	ODAM	OTUD7A	PAK1	PAK3
PAK7	PALB2	PAPD5	PARK2	PARP1	PARP2	PARP3	PARP4	PAX3	PAX5
PAX7	PAX8	PBRM1	PBX1	PCBP1	PCDH10	PDAP1	PDCD1	PDCD2L	PDE4DIP
PDGFB	PDGFRA	PDGFRB	PDK1	PDPK1	PDSS2	PER1	PGAP3	PGR	PHF6
PHOX2B	PIK3C2B	PIK3C2G	PIK3C3	PIK3CA	PIK3CB	PIK3CD	PIK3CG	PIK3R1	PIK3R2
PIK3R3	PIM1	PKHD1	PLAG1	PLCG1	PLCG2	PLEKHG5	PLK2	PMAIP1	PML
PMS1	PMS2	PNRC1	POLD1	POLE	POLQ	PORCN	POT1	POU2AF1	POU2F2
POU5F1	PPARG	PPM1D	PPP2R1A	PPP6C	PRDM1	PRKAR1A	PRKDC	PRLR	PRPF40B
PRSS8	PSIP1	PTCH1	PTEN	PTGS2	PTK2B	PTPN11	PTPRB	PTPRC	PTPRD
PTPRS	PTPRT	QKI	RAB40A	RAC1	RAD1	RAD21	RAD50	RAD51	RAD51B
RAD51C	RAD51D	RAD52	RAD54L	RAF1	RALGDS	RALY	RARA	RASA1	RB1
RBM10	RBMX	RECQL4	REL	RET	RFWD2	RHBDF2	RHEB	RHOA	RHOH
RICTOR	RIT1	RNASEL	RNF2	RNF213	RNF43	ROS1	RPA1	RPL10	RPL22

RPL5	RPS14	RPS15	RPS2	RPS6KA2	RPS6KA4	RPS6KB2	RPTOR	RRM1	RUNX1
RUNX1T1	RUNX3	RXRA	RYBP	SAMD9	SBDS	SDHA	SDHAF2	SDHB	SDHC
SDHD	SEPT9	SERPINB13	SETBP1	SETD2	SETDB1	SF1	SF3B1	SGK1	SH2B3
SH2D1A	SHQ1	SIN3A	SIRPA	SIRT4	SKP2	SLC19A1	SLC22A2	SLCO1B3	SMAD2
SMAD3	SMAD4	SMARCA4	SMARCB1	SMARCD1	SMARCE1	SMC1A	SMC3	SMO	SMUG1
SNX25	SOCS1	SOD2	SOS1	SOX10	SOX11	SOX17	SOX2	SOX9	SPEN
SPOP	SPRY4	SRC	SRSF2	SSX1	STAG2	STAT3	STAT4	STAT5B	STK11
STK19	STK36	STK38	STK40	STX2	SUFU	SULT1A1	SUZ12	SYK	SYNE1
SZRD1	TAF1	TAF1L	TAL1	TBC1D12	TBL1XR1	TBX22	TBX3	TCEB1	TCF12
TCF3	TCF4	TCF7L1	TCF7L2	TCL1A	TERT	TET1	TET2	TFE3	TFG
TGFBR1	TGFBR2	TGM7	THBS1	TIMM17A	TIMP3	TIPARP	TLK2	TLR4	TLX1
TMEM127	TMPRSS2	TNF	TNFAIP3	TNFRSF14	TNK2	TOP1	TP53	TP53BP1	TP63
TPMT	TPR	TPX2	TRAF3	TRAF7	TRIM24	TRIM33	TRIP11	TRRAP	TSC1
TSC2	TSHR	TSHZ2	TSHZ3	TYMS	TYR	U2AF1	U2AF2	UBR5	UGT1A1
UMPS	USP9X	VANGL2	VEZF1	VHL	VTCN1	WAC	WAS	WASF3	WHSC1
WISP3	WNK1	WRN	WT1	XIAP	XPA	XPC	XPO1	XRCC2	XRCC3
YAP1	YES1	ZFHX3	ZNF217	ZNF384	ZNF521	ZNF668	ZNF703	ZRANB3	ZRSR2

Cross-referencing with microarray probe information, 814 genes were detectable among the SCC-820 gene data set, and a total of 879 probes were selected, since some genes had multiple probes recognizing splice variants. The six genes whose probes were not present in the microarray were *MIR142, NBPF1, RAB40A, SULT1A1, UGT1A1*, and *PRPF40B.*

Table S3. Relationship between cancer stage and highly recurrent oncogenes amplified with overexpression in specific tumors.

Gene	Tumor type	Number of amplification/overexpression samples				Fisher's exact test
		Positive		Negative		
		Stage I, II	Stage III, IIV	Stage I, II	Stage III, IIV	*P*-value
MYC	Colorectal	45	77	122	248	0.4417
MYCN	Colorectal	1	11	166	314	0.0672
	Liver	8	1	50	28	0.2615
MET	Colorectal	33	57	134	268	0.5408
HIST1H3B	Colorectal	4	17	163	308	0.1637
	Lung	21	4	232	20	0.2502
	Liver	10	6	48	23	0.7717
	Breast	7	1	52	8	1.0000
EZH2	Colorectal	9	23	158	302	0.5648
	Lung	16	2	237	22	0.6609
	Liver	5	2	53	27	1.0000
	Breast	4	1	55	8	0.5197

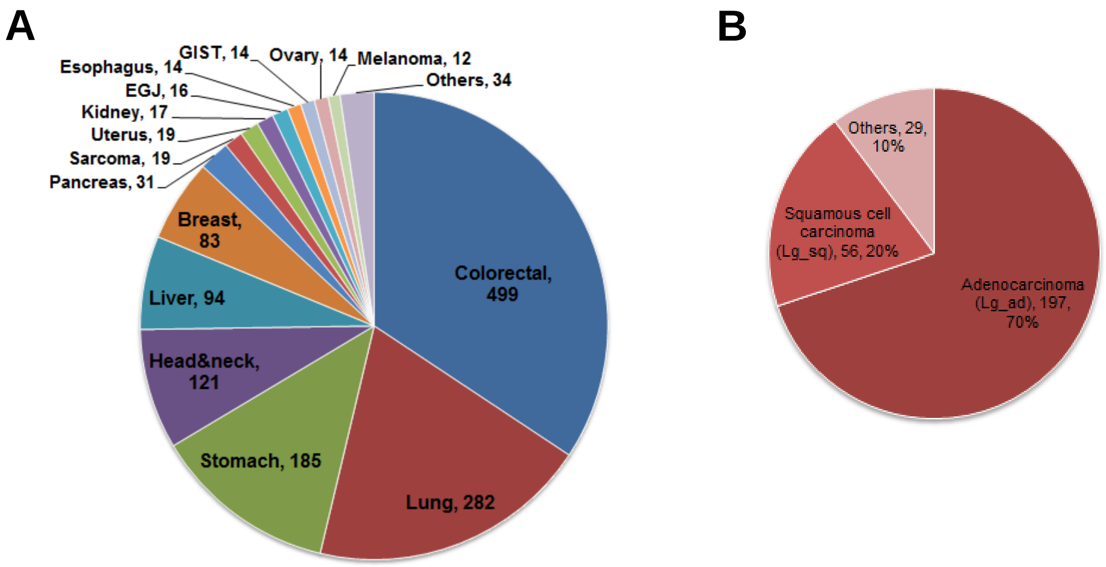

Figure S1. Classification of 1,454 tumor samples. (A) All samples classified by tumor type. **(B)** Lung cancer samples classified by tumor subtype including adenocarcinoma, squamous cell carcinoma, and others.

Overexpression frequency

0 100 (%)

FC≥10																5≤FC<10														
Colorectal	Lung	Stomach	Head&neck	Liver	Breast	Pancreas	Sarcoma	Uterus	Kidney	EGJ	Esophagus	GIST	Ovary	Melanoma		Colorectal	Lung	Stomach	Head&neck	Liver	Breast	Pancreas	Sarcoma	Uterus	Kidney	EGJ	Esophagus	GIST	Ovary	Melanoma
4	11	3	2	30	12	10	0	26	0	13	0	7	29	8	MYCN	11	8	4	4	14	8	16	16	32	0	0	0	7	14	17
9	0	1	0	3	1	0	0	0	24	0	0	0	0	0	MYC	29	0	3	2	1	0	0	11	0	24	6	0	0	0	0
5	7	1	6	3	18	6	0	32	18	6	0	0	21	0	MYCL v.1/2	11	6	2	7	6	22	10	5	26	0	0	7	0	0	8
11	1	8	10	0	1	3	0	11	0	13	36	7	0	0	NKX2-1	2	1	2	7	0	2	0	0	0	0	6	7	0	0	8
0	6	6	5	1	4	19	11	5	0	0	14	0	7	0	LMO1	0	9	4	2	2	7	0	0	0	12	0	14	0	7	0
0	1	1	2	0	0	0	0	0	0	6	0	0	0	0	SKP2	0	8	6	10	2	0	3	11	0	0	25	7	0	0	0
0	0	1	1	0	0	3	42	0	0	0	7	0	0	8	CCND1	2	0	1	2	1	4	3	11	0	18	0	7	7	7	0
0	1	1	0	0	1	0	11	0	0	0	0	7	0	0	MDM2	0	2	2	1	0	2	0	5	0	0	0	0	0	14	17
0	0	0	0	0	0	0	0	0	0	0	0	0	0	0	NCOA3	0	0	0	1	2	0	0	0	0	0	0	0	0	7	0
0	0	0	0	0	0	0	0	0	0	0	0	0	0	0	MYCL v.3	0	0	0	0	1	0	0	0	0	0	0	0	0	0	0
0	0	0	0	0	0	0	0	0	0	0	0	0	0	0	MDM4	0	0	0	0	0	0	0	0	0	0	0	0	0	0	0
16	18	5	17	24	25	3	79	42	6	19	14	0	0	8	HIST1H3B	7	19	20	9	27	33	16	5	26	29	31	21	7	36	17
17	8	2	7	4	11	0	26	26	0	6	0	0	14	0	EZH2	6	17	11	12	17	39	3	21	47	24	19	14	7	7	8
13	3	4	13	0	24	19	16	11	6	6	21	0	36	8	FGFR3	5	10	4	3	1	11	10	11	21	0	13	0	0	7	0
7	2	2	6	0	0	3	0	0	0	0	7	0	21	25	MET	18	4	1	20	0	0	0	0	16	12	6	14	0	14	8
5	1	1	2	2	1	0	5	5	6	13	7	7	7	0	CARD11	13	7	5	17	2	10	10	0	5	6	13	7	0	0	17
1	11	9	30	0	4	0	26	11	0	13	36	14	21	17	FOXL2	1	4	2	13	0	1	3	0	5	0	6	14	0	14	0
1	0	0	17	1	1	6	42	5	24	0	0	36	0	0	JAK3	7	1	4	17	4	5	3	26	5	18	13	0	21	21	25
0	5	3	7	4	23	0	0	0	0	0	14	0	0	0	RET v.2	0	4	4	12	1	14	3	5	0	6	0	7	0	0	0
0	1	1	8	2	22	0	5	5	0	0	7	0	0	8	FLT3	2	0	3	12	3	22	3	11	5	18	6	0	0	0	0
0	2	4	7	1	8	10	16	0	0	0	7	0	7	0	FGFR2	0	3	2	2	1	12	10	11	16	0	6	7	0	21	0
0	7	1	2	1	1	0	16	0	0	0	0	0	7	42	ALK	0	6	1	2	1	1	6	5	5	0	0	7	0	14	8
1	3	1	4	1	1	0	0	0	0	0	0	0	0	0	EGFR v.3	3	6	1	7	0	0	10	0	0	0	0	29	0	0	0
1	1	3	2	0	17	0	0	5	0	13	0	0	0	0	ERBB2	1	2	1	0	1	17	10	0	0	0	6	0	0	7	0
0	1	0	1	0	2	0	0	21	0	0	0	0	7	0	IDH2	0	10	0	1	0	10	0	0	21	0	0	0	0	14	0
0	0	1	2	6	5	3	5	0	12	0	0	100	0	17	KIT	0	1	1	2	5	4	6	21	0	0	0	0	0	0	17
0	3	1	6	0	1	0	0	0	0	0	7	0	0	0	EGFR v.1	0	6	1	4	0	0	6	0	0	18	0	7	0	0	0
0	1	1	0	1	0	0	5	0	0	0	0	21	0	0	SMO	1	4	1	1	5	0	0	21	0	0	0	0	29	0	8
0	3	1	1	0	11	0	0	0	0	0	0	0	0	0	RET v.4	0	0	1	1	0	14	0	0	0	0	0	0	0	0	0
0	0	0	2	0	0	6	21	0	6	0	0	0	0	0	CSF1R	1	0	1	5	0	0	6	26	0	24	0	0	0	0	8
1	2	1	1	0	0	0	0	0	0	0	0	0	0	8	TSHR	1	2	1	1	3	1	0	0	5	0	0	0	0	0	0
0	0	0	2	0	0	0	0	0	0	0	0	50	0	0	AR	0	1	2	2	0	2	3	0	0	0	0	0	21	0	0
0	0	0	0	0	0	0	0	0	0	0	0	0	0	0	IDH1	3	1	2	3	0	0	0	16	5	0	0	0	0	0	0
0	1	0	1	0	1	0	0	0	0	0	0	21	0	0	BCL2 v.α	0	3	1	0	1	0	0	0	0	12	0	0	21	0	8
0	0	0	0	0	0	0	5	0	0	0	0	0	0	0	DNMT1	1	0	1	3	3	1	0	37	5	0	0	0	0	0	8
0	0	1	0	0	0	0	0	0	0	0	0	0	0	0	DNMT3A v.1-3	0	3	1	1	2	2	3	5	5	0	0	0	0	0	0
0	0	0	0	0	1	0	0	0	0	0	0	0	0	0	BCL2 v.α/β	0	3	0	2	0	0	0	0	0	12	0	0	29	0	0
0	0	1	0	0	1	0	0	0	0	0	0	0	0	0	GATA2	1	0	2	2	4	0	0	0	0	0	0	0	0	0	0
0	0	1	1	1	0	3	0	0	0	0	0	0	0	0	MPL	0	0	0	1	3	0	6	0	0	6	0	0	7	7	0
0	0	0	2	0	1	0	0	5	0	0	0	0	0	0	NFE2L2	2	1	0	0	0	1	0	0	0	0	0	0	0	0	0
0	0	0	0	0	0	0	0	0	0	0	0	0	0	0	HRAS	0	2	1	5	1	0	0	0	0	6	0	0	0	0	0
0	0	0	0	1	0	0	0	0	0	0	0	0	0	0	KLF4	1	0	0	0	2	1	3	5	0	6	0	0	0	0	0
0	0	0	0	0	0	3	0	0	0	0	0	29	0	0	PDGFRA	0	0	1	0	1	0	6	11	0	0	0	0	14	0	0
0	0	0	1	0	0	0	5	0	0	0	0	0	0	0	MYD88	0	0	0	2	0	0	0	26	0	0	0	0	0	0	0
0	0	1	1	0	0	0	0	0	0	6	0	0	0	0	JAK2	0	0	1	2	0	0	3	0	0	0	0	0	0	0	0
0	0	0	0	0	0	0	0	0	0	0	0	0	0	0	NRAS	0	0	1	5	0	0	0	0	0	0	0	0	0	0	0
0	0	1	0	0	1	0	0	0	0	0	0	0	0	0	KRAS	0	0	0	0	0	0	0	0	0	0	0	0	0	0	0
0	0	0	0	0	0	0	0	0	0	0	0	0	0	0	SETBP1 v.1	0	0	0	0	0	0	3	0	0	0	0	0	21	0	0
0	0	0	0	0	0	0	0	0	0	0	0	0	0	0	ABL1	0	0	0	0	0	0	0	11	0	0	0	0	0	0	0
0	0	0	0	0	0	0	0	0	0	0	0	0	0	0	CBL	0	0	0	1	0	0	3	0	0	0	0	0	0	0	0
0	0	0	0	0	0	0	0	0	0	0	0	0	0	0	CRLF2	0	0	0	0	0	0	0	0	0	0	0	0	0	0	0
0	0	0	0	0	0	0	0	0	0	0	0	0	0	0	CTNNB1	0	0	1	0	0	0	0	0	0	0	0	0	0	0	0
0	0	0	0	0	0	0	0	0	0	0	0	0	0	0	GNAQ	0	0	0	1	0	0	0	0	5	0	0	0	0	0	0
0	0	0	0	0	0	0	0	0	0	0	0	0	0	0	PIK3CA	0	0	0	0	0	0	0	0	0	0	0	0	0	0	0
0	0	0	0	0	0	0	0	0	0	0	0	0	0	0	SETBP1 v.2	0	0	0	0	1	0	0	0	0	0	0	0	7	0	0
0	0	0	0	0	0	0	0	0	0	0	0	0	0	0	DNMT3A v.4	0	0	1	0	0	0	0	0	0	0	0	0	0	0	0
0	0	0	0	0	0	0	0	0	0	0	0	0	0	0	GNAS	0	0	0	0	0	0	0	0	0	0	0	0	0	0	0
0	0	0	0	0	0	0	0	0	0	0	0	0	0	0	SPOP	0	0	0	1	0	0	0	0	0	0	0	0	0	0	0

Figure S2. Frequency of elevated expression of oncogenes in individual tumor types. Frequency of strong (fold change ≥ 10) and moderate (5 ≤ fold change < 10) overexpression is indicated on the left and right, respectively, as a heat map (ranging 0%–100%). The frequencies of amplification-, and mutation-based, oncogenes are shown in the upper and lower panels, respectively.

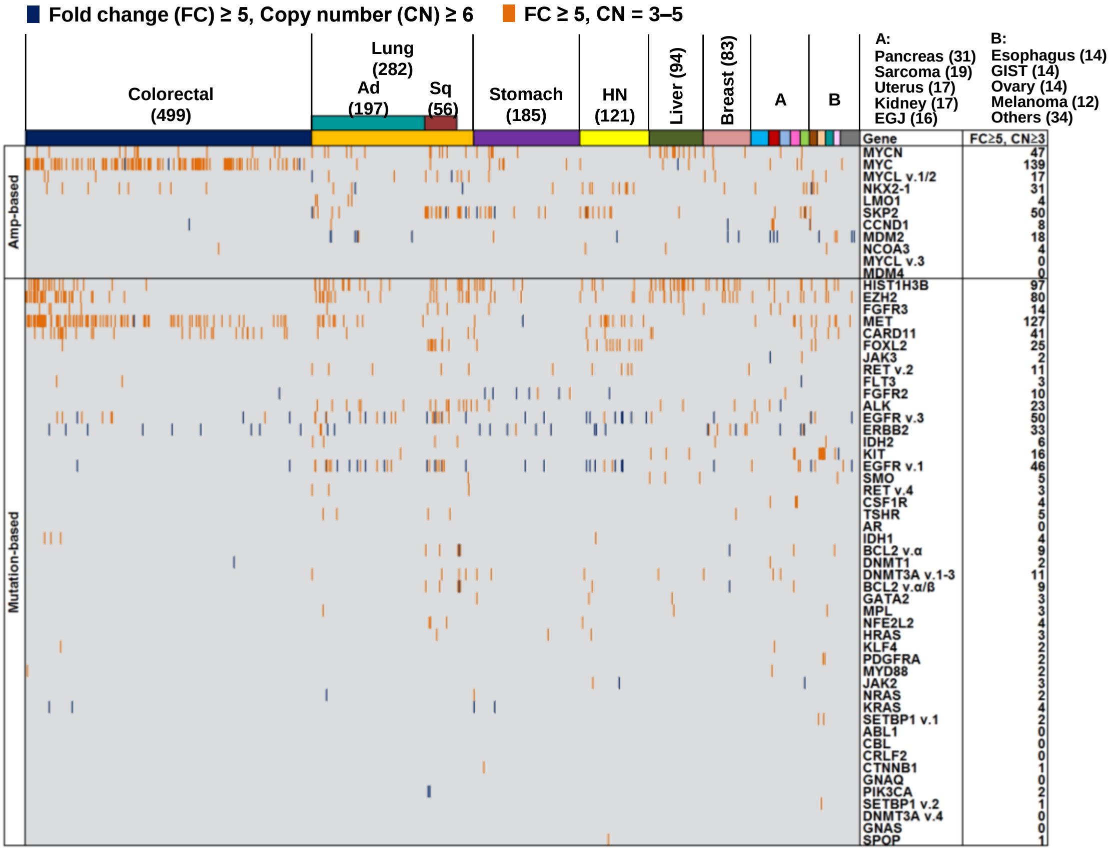

Figure S3. Elevated gene expression with copy number gain of 64 oncogenes in 1,454 solid tumors. Genes overexpressed ≥ 5-fold with high (copy number ≥ 6) and moderate (copy number 3–5) genomic amplification are indicated by dark blue and orange bars, respectively. Genes are divided into two groups; amplification- and mutation-based oncogenes, in the upper and lower panels, respectively. The numbers on the right indicate the total number of samples with each gene overexpressed ≥ 5-fold and genomic copy number ≥ 3. As indicated at the top, samples are arranged by tumor type, including tumors from colorectal, lung, stomach, head and neck (HN), liver, breast, pancreas, sarcoma, uterus, kidney, esophagogastric junction (EGJ), esophagus, gastrointestinal stromal tumor (GIST), ovary, melanoma, and other types of tumors. The number of samples of each type of tumor is indicated in parentheses.

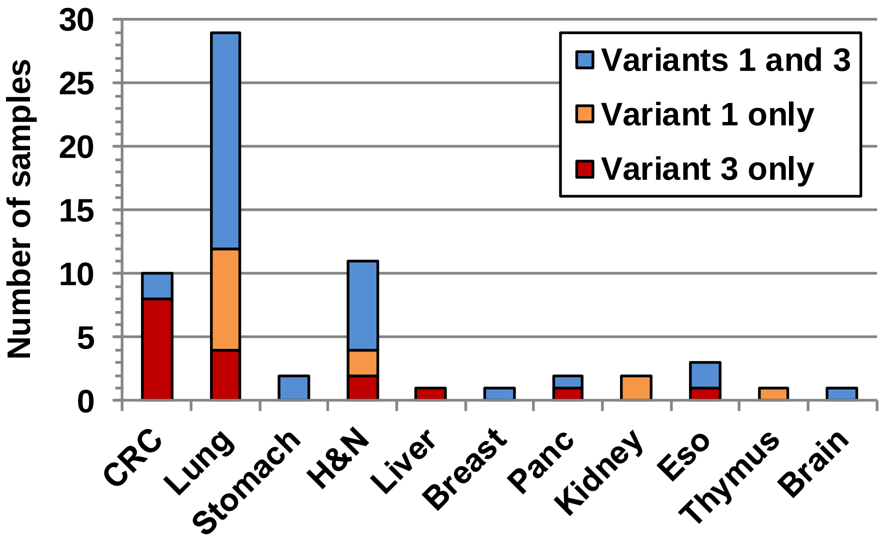

Figure S4. Number of samples exhibiting overexpression and amplification of EGFR variants 1 and 3 in individual cancer types. The total number of samples exhibiting EGFR overexpression (≥5-fold) and amplification (copy number ≥ 3) was 46 and 50 for variants 1 and 3, respectively. Among these, there were 33 samples in which both variants were overexpressed and amplified, and 11 and 17 samples in which only variant 1 or 3 was overexpressed and amplified, respectively. As indicated at the bottom, samples are arranged by tumor type, including tumors from colorectal (CRC), lung, stomach, head and neck (H&N), liver, breast, pancreas (Panc), kidney, esophagus (Eso), thymus, and brain.

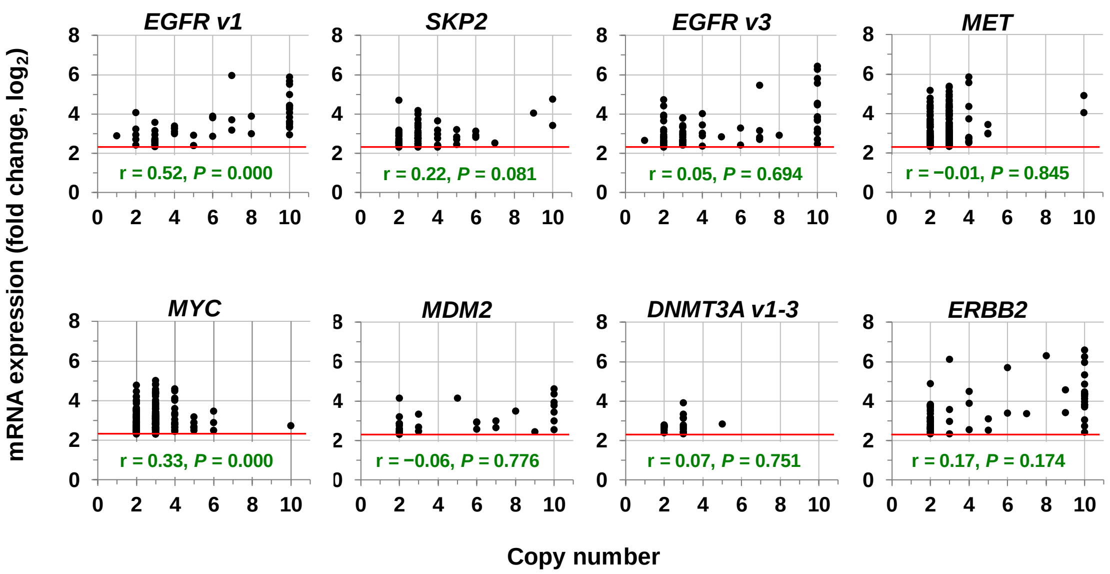

Figure S5. Copy number and gene expression of eight oncogenes exhibiting high frequency of overexpression with amplification. Genes amplified and overexpressed with frequency ≥ 50% include *EGFR* variant 1 (88%), *SKP2* (76%), *EGFR* variant 3 (70%), *MET* (65%), *MYC* (64%), *MDM2* (62%), *DNMT3A* variant 1-3 (52%), and *ERBB2* (50%) as listed in Table 1. Pearson's correlation coefficient (r with *P*-value) between copy number and mRNA expression is indicated at the bottom. Red horizontal bars indicate 5-fold on the log_2 scale.

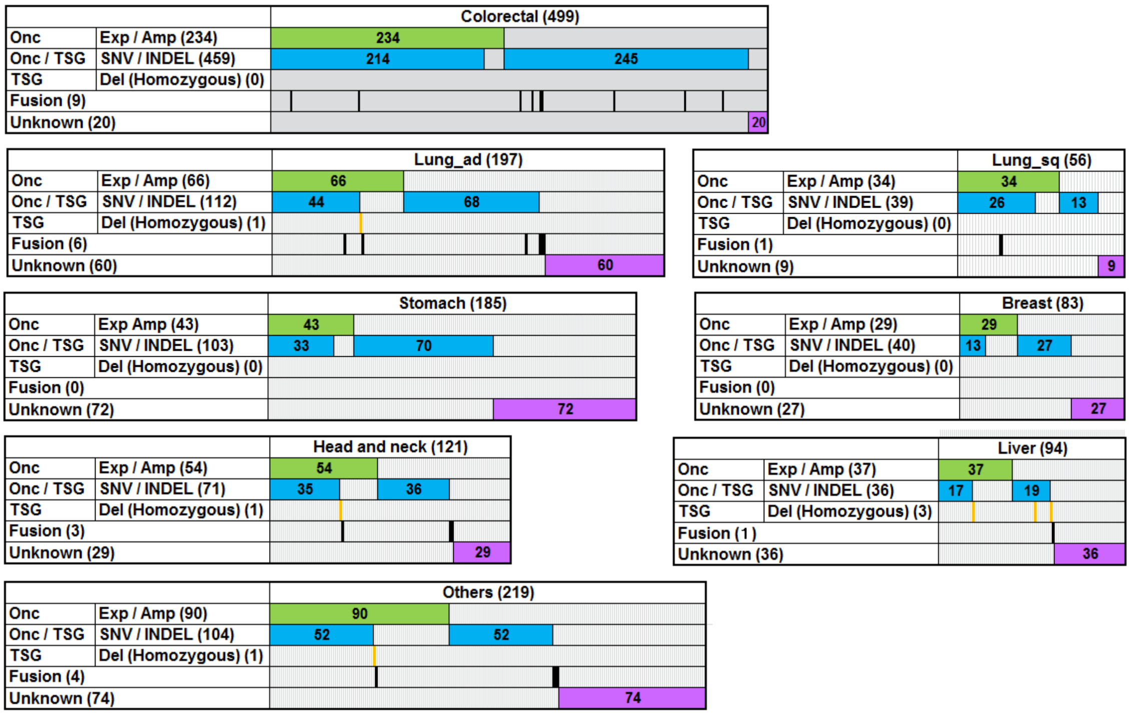

Figure S6. Driver mutations, fusions, and genomic amplification in 1,454 solid tumors. Somatic structural alterations in individual tumor types are categorized into five types, as follows: overexpression with gene amplification in the 64 oncogenes (Onc, Exp/Amp); mutations, insertions, and deletions in the 138 driver genes (Onc/TSG, SNV/INDEL); homozygous deletions in the 74 tumor suppressor genes (TSG, Del); fusions of the 491 genes; and no alterations (Unknown). The driver effects of the 74 TSGs were assigned with the assumption of haploinsufficiency exertion of dominant-negative effects.

A

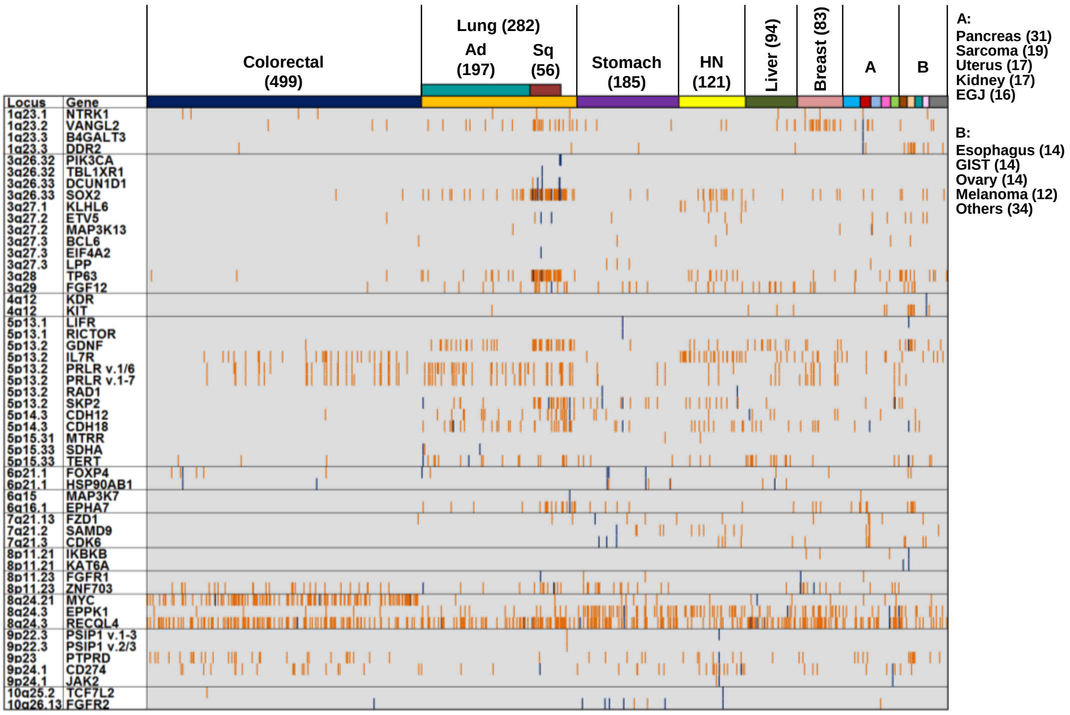
Fold change (FC) ≥ 5, Copy number (CN) ≥ 6
FC ≥ 5, CN = 3–5
Colorectal (499)
Lung (282)
Ad (197)
Sq (56)
Stomach (185)
HN (121)
Liver (94)
Breast (83)
A
B
Locus
Gene
A:
Pancreas (31)
Sarcoma (19)
Uterus (17)
Kidney (17)
EGJ (16)
B:
Esophagus (14)
GIST (14)
Ovary (14)
Melanoma (12)
Others (34)

B

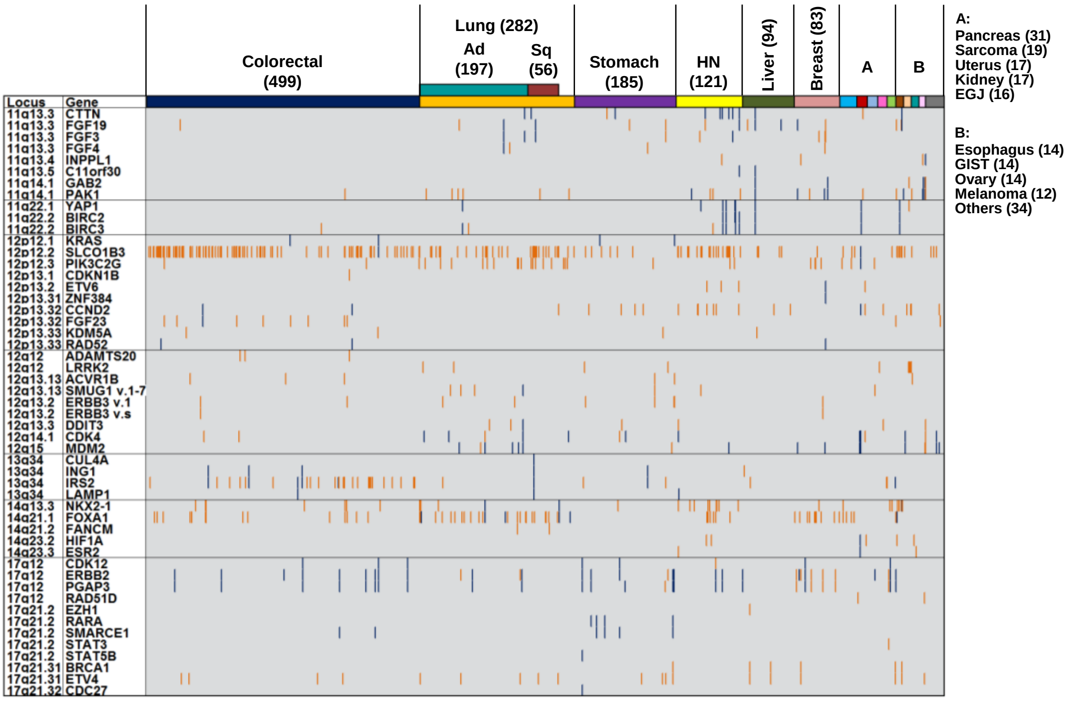
Fold change (FC) ≥ 5, Copy number (CN) ≥ 6
FC ≥ 5, CN = 3–5
Colorectal (499)
Lung (282)
Ad (197)
Sq (56)
Stomach (185)
HN (121)
Liver (94)
Breast (83)
A
B
Locus
Gene
A:
Pancreas (31)
Sarcoma (19)
Uterus (17)
Kidney (17)
EGJ (16)
B:
Esophagus (14)
GIST (14)
Ovary (14)
Melanoma (12)
Others (34)

C

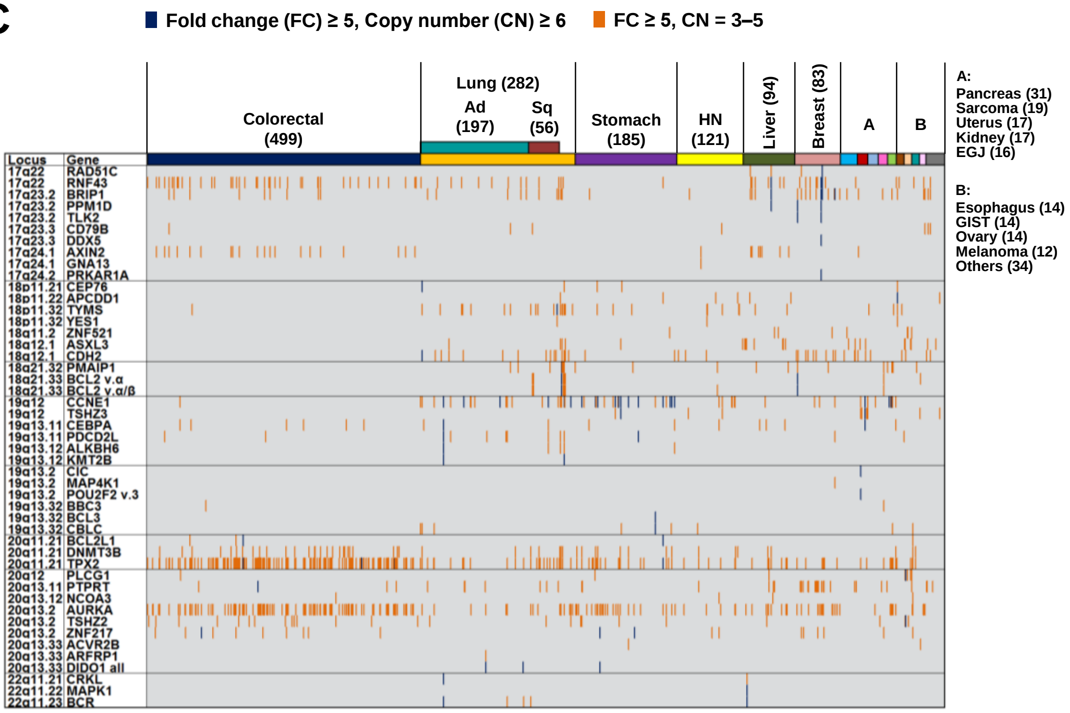

Figure S7. Overexpressed and amplified chromosomal loci containing multiple cancer-related genes in 1,454 solid tumors. Genes with overexpression and amplification are clustered by chromosomal locus, as indicated on the left. Genes overexpressed ≥ 5-fold with high (copy number ≥ 6) and moderate (copy number 3–5) genomic amplification are indicated by dark blue and orange bars, respectively. As indicated at the top, samples are arranged by tumor type, including tumors from colorectal, lung, stomach, head and neck (HN), liver, breast, pancreas, sarcoma, uterus, kidney, esophagogastric junction (EGJ), esophagus, gastrointestinal stromal tumor (GIST), ovary, melanoma, and other types of tumors. The number of samples of each type of tumor is indicated in parentheses.
